# Characterizing the degradation of cannabidiol in an e-liquid formulation

**DOI:** 10.1038/s41598-022-23910-6

**Published:** 2022-11-21

**Authors:** Adrián Schwarzenberg, Harry Carpenter, Christopher Wright, Omer Bayazeid, Michał Brokl

**Affiliations:** B.A.T. (Investments) Limited, Southampton, SO15 8TL UK

**Keywords:** Analytical chemistry, Mass spectrometry, Natural products

## Abstract

The reported characteristics of cannabidiol (CBD) have encouraged significant growth in commercial CBD products. There is limited information on the stability of CBD and some researchers have noted significant reductions of CBD in products. In this study, the chemical profiles of plant-based and chemically synthesized CBD in a prototype e-liquid formulation were assessed during 4 weeks of storage under varying conditions. Samples were analysed on days 1, 8, 15, 22, and 29 by untargeted analysis using ultra-high performance liquid chromatography—trapped ion mobility–time-of-flight mass spectrometry (UHPLC-TIMS-TOF-MS). On day 1, analysis of plant-based and synthetic CBD formulations showed small differences in their composition, with plant-based CBD e-liquid containing trace levels of a higher number of phytocannabinoid-related impurities. Storage for 4 weeks under stress (40 °C, 75% relative humidity, dark) and ambient (25 °C, 60% relative humidity, daylight) conditions led to increases in the number and abundance of cannabinoid-related degradation products, including cannabielsoin (CBE) and CBD-hydroxyquinone (HU-331), which are products of the oxidation of CBD, and other unidentified cannabinoid-related compounds. The unidentified cannabinoid-related compounds were probed by accurate mass measurement and MS^2^ fragmentation but could not be matched using a mass spectral library derived from 39 commercially available cannabinoid reference standards. Based on elemental composition and MS^2^ fragmentation patterns, the unidentified cannabinoid-related compounds were classified as hydroxy-CBE, hydroxy-CBD, and dihydroxy-CBD. The analysis of e-liquid formulations protected from light and stored at 4 °C for 4 weeks indicated only very small increases in CBD oxidation products. The results indicate that CBD degrades in e-liquid solution at ambient temperature in dark and light to form potentially undesirable products, including cannabielsoin and cannabidiol hydroxyquinone.

## Introduction

Phytocannabinoids are a class of compounds that occur naturally in the cannabis plant (*Cannabis sativa*). More than 100 phytocannabinoids have been identified, of which Δ9-tetrahydrocannabinol (THC) and cannabidiol (CBD) have been extensively documented^1^. In recent years, the non-psychoactive phytocannabinoid CBD has been widely studied and its properties have led to a rapid increase of its use in a wide array of consumer products^2^.

E-cigarettes were originally developed as an alternative nicotine delivery method to conventional cigarettes in order to avoid the health risks associated with inhalation of cigarette smoke. In a standard e-cigarette, nicotine is dissolved in an “e-liquid” comprising propylene glycol (PG) and vegetable glycerin (VG), which is then vapourised to an inhalable aerosol by a heating element. The e-cigarette format has also been used to deliver CBD at concentrations ranging from 10 to 200 mg per mL of e-liquid in the UK market.

E-cigarettes and e-liquids are highly regulated, with manufacturers required to supply a list of ingredients and quantities thereof^3,4^. The CBD used in e-liquids may be either extracted from hemp, for example by supercritical carbon dioxide extraction^5^, or manufactured synthetically. Plant-derived CBD varies in grade from ‘full-spectrum’, containing multiple other cannabinoids, flavonoids and other naturally occurring compounds, to CBD isolate, which in theory contains no other cannabinoids, terpenes or flavonoids^6^, but in practice may contain traces of these compounds^7^. In this regard, a method that can detect other cannabinoids present in CBD products can enable the characterisation of CBD from different sources.

Because it is polyphenolic in nature and vulnerable to oxidation and/or photodegradation, CBD may be unstable in some formulations^8–10^. Daylight exposure has been shown to reduce CBD stability in cannabis extracts^11,12^ and to decrease CBD levels in resin^12^. Temperature also affects the degradation of CBD in solution^9,13^. CBD oxidizes on exposure to air, forming its hydroxyquinone (HU-331) and potentially other products^14–16^. While published studies have demonstrated, using high-performance liquid chromatography (HPLC)^8,9,13^, that other compounds are formed by the breakdown of CBD their identities have not been confirmed.

The purpose of this study was to identify substances formed by the degradation of CBD in an e-liquid using ultra-HPLC (UHPLC) coupled with trapped ion mobility–time-of-flight–MS (TIMS-TOF-MS)^17,18^, an advanced analytical technique with high-resolving power^18,19^ that has been recently applied to plant metabolomics^17^. To support identification, an in-house mass spectral library was developed comprising chromatographic and MS data obtained from the analysis of 39 reference phytocannabinoid compounds. The use of TIMS increased the diagnostic information with which to accurately identify phytocannabinoids and facilitated a comparison of the impurities present in CBD from different sources (plant-based and synthetic) including cannabidivarin (CBDV), cannabidibutol (CBDB) and cannabidiphorol (CBDP). The formation of CBD degradation products, including cannabielsoin (CBE), hydroxy-cannabidiol (OH-CBD), their positional isomers and an unidentified CBE-like cannabinoid, was evaluated for prototype CBD e-liquids stored under different conditions of temperature, humidity, and light exposure.

## Results

### Optimisation of UHPLC-TIMS-TOF-MS for cannabinoid analysis

**Figure 1:**
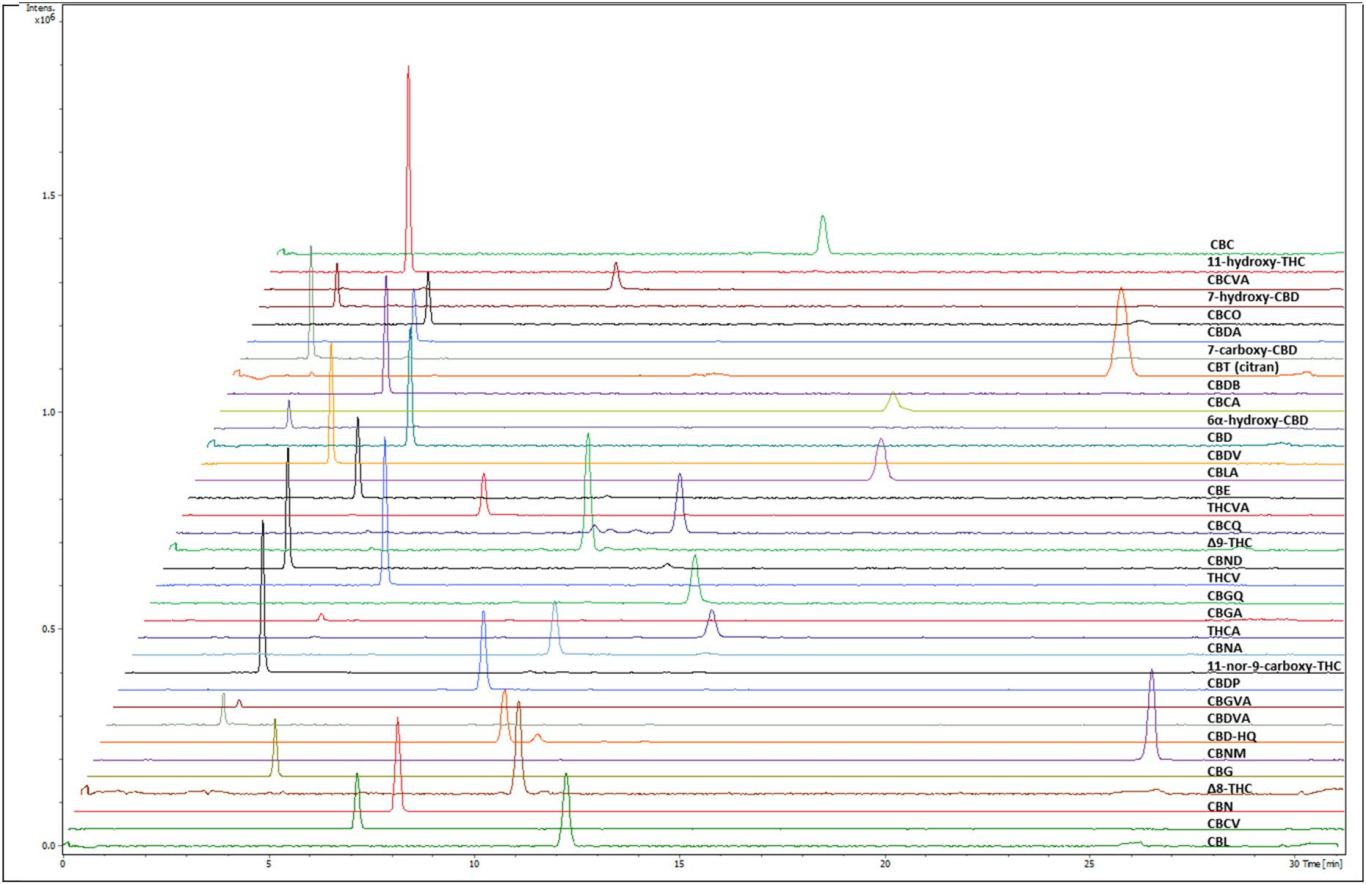
Optimised separation of 35 commercially available cannabinoids by UHPLC-TIMS-TOF-MS. From top to bottom: cannabichromene (CBC), 11-hydroxy-Δ9-tetrahydrocannabinol (11-OH-Δ9-THC), cannabichromevarinic acid (CBCVA), 7-hydroxy-cannabidiol (7-hydroxy-CBD), cannabichromeorcin (CBCO), cannabidiolic acid (CBDA), 7-carboxy-cannabidiol (7-COOH-CBD), cannabicitran (CBT), cannabidibutol (CBDB), cannabichromenic acid (CBCA), 6-α-hydroxy-cannabidiol (6-α-OH-CBD), cannabidiol (CBD), cannabidivarin (CBDV), cannabicyclolic acid (CBLA), cannabielsoin (CBE), tetrahydrocannabivarinic acid (THCVA), cannabichromenquinone (CBCQ), Δ9-tetrahydrocannabinol (Δ9-THC), cannabinodiol (CBND), tetrahydrocannabivarin (THCV), cannabigeroquinone (CBGQ), cannabigerolic acid (CBGA), Δ9-tetrahydrocannabinolic acid A (THCA), cannabinolic acid (CBNA), ( −)-11-nor-9-carboxy-Δ9-THC (11-COOH-Δ9-THC), cannabidiphorol (CBDP), cannabigerovarinic acid (CBGVA), cannabidivarinic acid (CBDVA), cannabidiol hydroxyquinone (CBD-HQ/HU-331), cannabinolmonomethlyether (CBNM), cannabigerol (CBG), Δ8-tetrahydrocannabinol (Δ8-THC), cannabinol (CBN), cannabichromevarin (CBCV) and cannabicyclol (CBL).

To analyse potential impurities and degradants present in CBD formulations, an UHPLC-TIMS-TOF-MS method was developed and optimized to separate and detect phytocannabinoids using 35 cannabinoid standards that were commercially available at the time (Fig. 1). 4 additional cannabinoid standards become available later and were also added to the library. Chromatographic, accurate mass, MS/MS and other data were collated to establish a reference mass spectral library. The average limit of quantification (LOQ) for the different cannabinoids was 10 ng/mL.

Samples were analysed by TIMS-TOF-MS in both positive and negative ionisation modes using an electrospray ionisation source (ESI). However, the negative ionisation mode did not provide much information on the compounds of interest and therefore all the MS data presented in this manuscript were from positive ionisation mode.

### Formulation and storage of CBD e-liquids

To characterise impurities associated with plant-based and synthetic CBD in e-liquid formulations stored under different conditions, two unflavoured, non-commercial CBD e-liquids were prepared by adding either CBD isolate (60 mg/mL) or synthetic CBD (60 mg/mL) to a mixture of pharmaceutical-grade propylene glycol (70%) and pharmaceutical-grade glycerol (30%). All CBD materials tested in this study were in compliance with the UK regulation. Each e-liquid was divided into multiple vials for storage under four different conditions (n = 5 each): (1) exposure to light/dark cycles at ambient temperature (25 °C) and ambient humidity (60% RH); (2) storage in the dark at ambient temperature and humidity; (3) dark storage at 4 °C (refrigerated conditions); and (4) dark storage at 40 °C and 75% RH (stressed conditions). Condition 4 was intended to stress the CBD liquid samples to generate significant levels of degradation products for identification. Aliquots of the stored samples were taken and diluted with acetonitrile to a final concentration of 100 µg/mL for cannabinoid analysis on day one and then at weekly intervals (at 8, 15, 22 and 29 days).

#### Initial cannabinoid profiling of CBD e-liquid

The first analysis was conducted one day after the preparation of the e-liquids to identify baseline CBD-related impurities and early degradation products. In total, eleven cannabinoid impurities/degradants were detected in the e-liquids. Of these, five were detected only in the isolate CBD e-liquid (cannabidihexol [CBDH], CBDP, 6α-OH-CBD, 7-OH-CBD, CBDV); one was detected only in the synthetic CBD e-liquid (CBDH isomer); and four were detected in both types of e-liquid (CBDB, OH-CBE, HU-331, HU-331-like) (Table 1).

**Table 1:** Cannabinoid impurities and degradants identified by UHPLC-TIMS-TOF-MS in CBD e-liquids detected 1 day after formulation into e-liquid.

RT (min)	Cannabinoid	*m/z*	Adduct	Elemental composition	Mass accuracy (Δppm)	ΔCCS_TIMS_ (%)^a^	Synthetic CBD	Isolate CBD	Validated^b^
1.84	6αOH-CBD	331.2265	[M + H]^+^	C_21_H_31_O_3_	0.7	0.1	No	Yes	Yes
2.03	7-OH CBD	331.2266	[M + H]^+^	C_21_H_31_O_3_	0.6	0.6	No	Yes	Yes
2.42	HU-331-like^c^	329.2170	[M + H]^+^	C_21_H_29_O_3_	1.1	0.3	Yes	Yes	No
2.76	OH-CBE^c^	347.2216	[M + H]^+^	C_21_H_31_O_4_	− 0.1	N/A	Yes	Yes	No
3.20	CBDV	287.2003	[M + H]^+^	C_19_H_27_O_2_	0.8	0.69	No	Yes	Yes
3.92	CBDB	301.2160	[M + H]^+^	C_20_H_29_O_2_	0.5	0.6	Yes	Yes	Yes
4.75	OH-CBE^c^	347.2216	[M + H]^+^	C_21_H_31_O_4_	0.2	N/A	Yes	Yes	No
6.56	CBDH	329.2473	[M + H]^+^	C_22_H_33_O_2_	0.4	0.2	No	Yes	Yes
8.95	CBDP	343.2632	[M + H]^+^	C_23_H_35_O_2_	0.1	0.6	No	Yes	Yes
9.65	CBDH isomer^c^	329.2472	[M + H]^+^	C_22_H_33_O_2_	0.9	N/A	Yes	No	No
9.92	HU-331	329.2109	[M + H]^+^	C_21_H_29_O_3_	–0.5	0.3	Yes	Yes	Yes

*CBDB* cannabidibutol; *CBDH* cannabidihexol; *CBDP* cannabidiphorol; *CBDV* cannabidivarin;CBD-HQ; *CBD* hydroxyquinone (HU-331); *CBE* cannabielsoin; *OH-CBD* hydroxy cannabidiol; *OH-CBE* hydroxy-cannabielsoin.

^a^ΔCCS_TIMS_ was estimated by comparing the CCS value with that obtained for the certified reference material (CRM) on the same instrument.

^b^Validated by matching to an analytical standard based on retention time, exact mass measurement, isotopic pattern, collisional cross section, and MS^2^ fragmentation.

^c^Putative identification.

**Figure 2:**
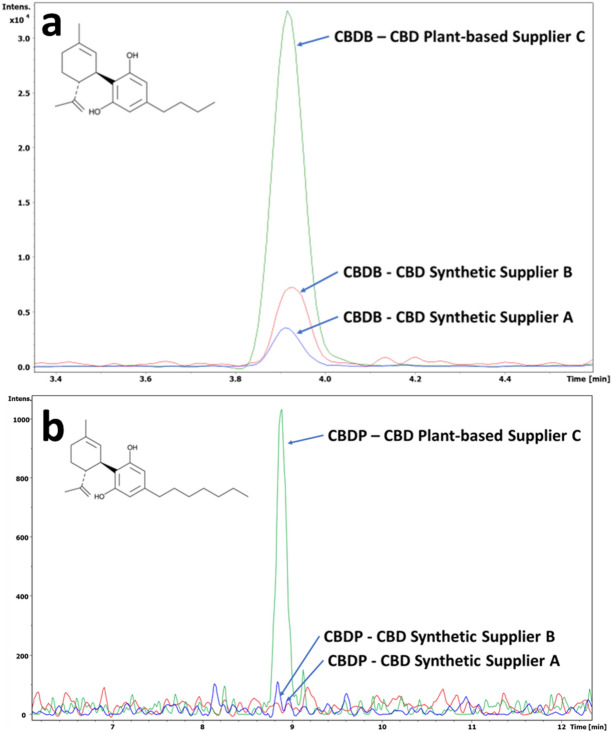
High-resolution extracted ion chromatogram of (**a**) CBDB [M + H]^+^ at *m/z* 301.2157 and (**b**) CBDP [M + H]^+^ at *m/z* 343.2632 of synthetic and plant-based CBD formulation samples.

Impurity profiling may be used to distinguish plant-based CBD from synthetic CBD because certain cannabinoids, such as CBDB and CBDV, are suggested to be present only in plant-derived CBD^7,20^. CBDB was detected in the e-liquid containing plant-based CBD, as previously reported^20^, but it was also found in the synthetic CBD liquid, although at lower abundance. To evaluate the presence of CBDB, synthetic CBD from different sources was formulated into e-liquids and analysed, including three different batches of CBD material from supplier A and one synthetic certified reference material (CRM) from supplier B. Monitoring the high-resolution extracted ion chromatogram (HRXIC) of CBDB at *m/z* 301.2157 (C_20_H_28_O_2_, Δppm 0.48) showed that this compound was present in all tested CBD samples (Fig. 2a) and was confirmed by comparison with an authentic analytical standard using five quality criteria (retention time, isotopic pattern, exact mass accuracy, collisional cross section [CCS_TIMS_] and MS^2^ fragmentation).

Although CBDB was present in formulations containing both synthetic and plant-based CBD, other cannabinoid impurities were more specific to the source of CBD. For example, CBDP was detected in plant-based CBD e-liquid (Fig. 2b) but was not detected in formulations containing synthetic CBD from supplier A or supplier B at the LOQ, which was estimated to be 5 ng/mL (at a signal-to-noise ratio of 10). Based on the calibration curve for this compound, its concentration in the plant-based CBD e-liquid formulation was ~ 5.9 µg/g. Despite the low intensity of the peak, the TIMS-TOF-MS detection gave a mass accuracy of  − 0.11 ppm, and close correspondence to the commercially available compound (ΔCCS_TIMS_ 0.6%). In addition, CBDH was identified at *m/z* 329.2473 (C_22_H_32_O_2_, Δppm 0.39) only in plant-based CBD e-liquid; in CBDH, a hexyl alkyl chain is attached to the resorcinol group instead of the pentyl alkyl chain found in CBD^21^. A peak that was putatively identified as a CBDH isomer was detected at *m/z* 329.2472 (C_22_H_32_O_2_, Δppm 0.89), but only in the synthetic CBD formulation (see Supplementary Fig. S1a). Initial tentative assignment of this compound as cannabidiol monomethyl ether (CBDM) or Δ9-tetrahydrocannabihexol (THCH) was rejected after comparison with an authentic analytical standard. The CBDH isomer had the same fragmentation pattern as CBDH and CBDM but different retention time. Unfortunately, the other isomer, Δ9-tetrahydrocannabidiol monomethyl ether (THCM), was not commercially available to enable confirmatory analysis. Putative identification of CBDH isomer was based on accurate mass measurement, CCS_TIMS_, and MS^2^ spectra (Supplementary Fig. S1b,c), although MS^2^ fragmentation did not allow differentiation of the two cannabinoids. Lastly, two isomeric hydroxylated CBD compounds at *m/z* 331.2260 were detected only in plant-based CBD e-liquid, namely 6α-OH-CBD and 7-OH-CBD, eluting at 1.83 min and 2.03 min, respectively (Supplementary Fig. S2).

Regarding degradants detected in both e-liquids, two isomeric peaks eluting at 2.76 min and 4.75 min with *m/z* 347.2216, a unique elemental composition of C_21_H_31_O_4_, and a calculated mass error (Δppm) of –0.07 ppm were observed (Supplementary Fig. S3). Both peaks were classified as hydroxy-cannabielsoin (OH-CBE) due to diagnostic fragment ions at *m/z* 205.1224 and *m/z* 135.0492. In addition, cannabidiol hydroxyquinone (HU-331), an established oxidation product of CBD^14^, was also identified and confirmed by comparison with an authentic standard.

The results of analysis demonstrate that the profiling of impurities/early degradants can indicate the source of CBD material. As previously reported^7,20^, CBDV was detected only in plant-based CBD e-liquid. Two other compounds, CBDH and CBDP, were also detected only in the plant-based CBD formulation (Fig. 2a,b). Conversely the CBDH isomer was detected only in synthetic CBD from supplier A and the synthetic CRM CBD from supplier B and thus could be used as a diagnostic compound to differentiate between the two CBD sources.

#### Identification of CBD breakdown compounds

As indicated above, analysis of the prototype e-liquids shortly after formulation (1 day) indicated the presence of cannabinoid impurities and/or oxidation products at very low levels (Table 1). After 8 days of storage under stressed conditions (40 °C/75% RH), several additional peaks in the UHPLC-TIMS-TOF-MS spectra of the e-liquids were observed. At the subsequent timepoints (8, 15, 22 and 29 days), the number of detected peaks remained the same, but their abundances increased over time (Supplementary Fig. S4, S5).

**Figure 3:**
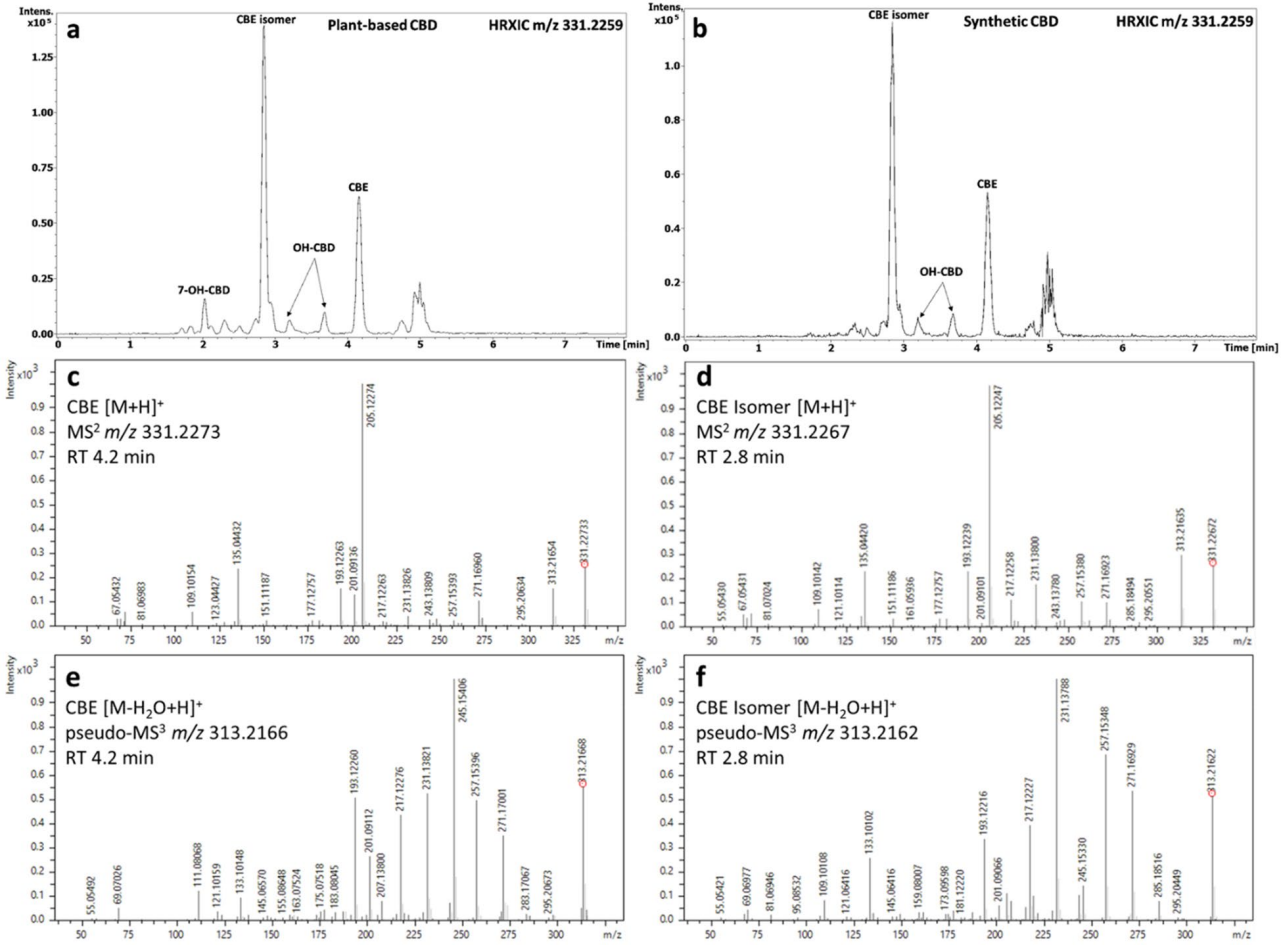
Representative high-resolution extracted ion chromatograms of CBE *m/z* 331.2259 in CBD e-liquid after 4 weeks of storage at 40 °C and 75% RH. (**a**) Plant-based CBD formulation; (**b**) synthetic CBD formulation. (**c**) MS^2^ fragmentation spectrum of CBE. (**d**) MS^2^ fragmentation of the *m/z* 331.2259 ion putatively identified as CBE isomer. (**e**) Pseudo-MS^3^ fragmentation of the [M-H_2_O + H]^+^ *m/z* 313.2166 ion of CBE. (**f**) Pseudo-MS^3^ fragmentation of the [M-H_2_O + H]^+^ *m/z* 313.2162 ion of the CBE isomer.

One of the main degradants in both plant-based and synthetic CBD e-liquid was identified as CBE ([M + H]^+^, *m/z* 331.2262; –0.7 Δppm; RT, 4.2 min). Interestingly, another major peak with the same mass and elemental composition as CBE was observed at a different retention time (RT, 2.8 min; Fig. 3a,b). To investigate the identity of this unknown peak, its MS^2^ fragmentation spectrum was compared with that of CBE, which showed characteristic ions at *m/z* 205.1224, *m/z* 135.0442 and *m/z* 109.1042. As observed in Fig. 3c,d, the unknown peak showed a similar fragmentation pattern and therefore was putatively identified as a CBE isomer.

Dual TIMS was used to perform parallel accumulation serial fragmentation (PASEF)^19,22,23^, in which the TIMS cell is synchronized with MS^2^ precursor selection, enabling the fragmentation of almost all detected ions. This allowed pseudo-MS^3^ fragmentation to be performed by selecting fragment ions produced in-source during ionisation. The CBE mass spectral data indicated a neutral loss of 18 Da, corresponding to loss of an H_2_O molecule, giving the ion [M–H_2_O + H]^+^ *m/z* 313.2166. Pseudo-MS^3^ fragmentation of this ion indicated that CBE can be differentiated from the ‘CBE isomer’ by changes in the intensity of ions at *m/z* 245.1540 and *m/z* 231.1378 (Fig. 3e,f). This difference may derive from the position of the OH group in the terpenyl moiety or other modification. It was not possible to identify the position of the OH group from the MS data.

Ion mobility measurement showed a slight difference in CCS_TIMS_ between CBE (186.0 Å^2^) and the CBE isomer (184.9 Å^2^), indicating that the two molecules have a similar structure. To confirm the structure of the CBE isomer, a one-year-old sample kept under light conditions was used to isolate the CBE isomer peak. The isolated CBE isomer was analysed by Nuclear Magnetic Resonance (NMR) spectroscopy. 1D and 2D NMR data showed the CBE isomer is not a structural isomer of CBE but a stereoisomer (Supplementary Fig. S6).

**Figure 4:**
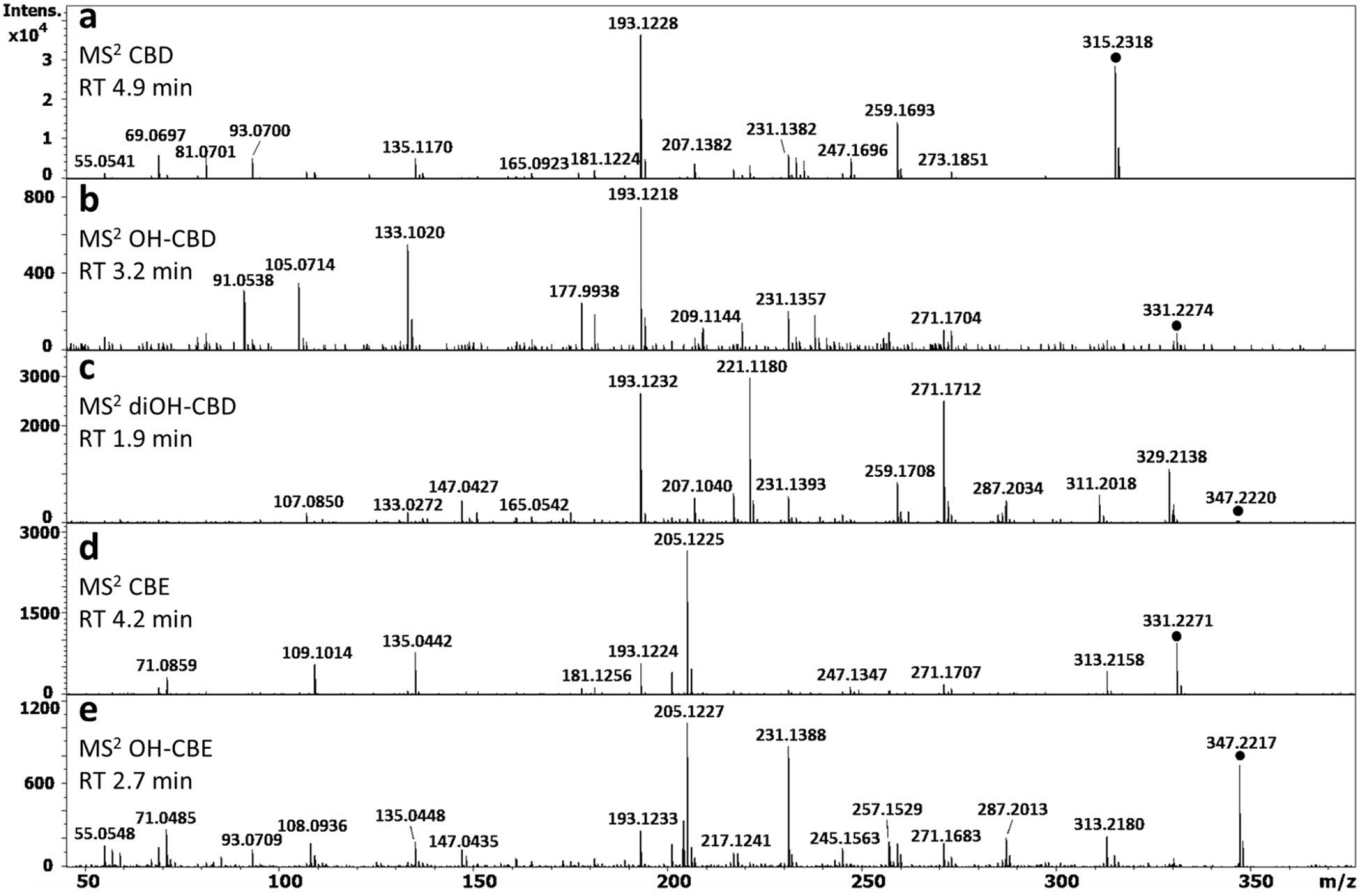
MS^2^ fragmentation spectrum of CBD at RT 4.99 min (**a**), OH-CBD at RT 3.2 min (**b**), diOH-CBD at RT 1.9 min (**c**), CBE at RT 4.2 min (**d**), and OH-CBE at RT 2.7 min (**e**).

Other products of CBD degradation were observed in e-liquid stored under different conditions, including HU-331 at *m/z* 329.2109. Interestingly, in the HRXIC of the *m/z* 329.2109 ion, HU-331 was observed at RT 9.91 min as expected, however, several peaks were also observed at different retention times (Supplementary Fig. S7). Some of which corresponded to in-source water loss from the *m/z* 347.2220 ion (diOH-CBD), which in turn gave a fragment ion at *m/z* 329.2109 (Fig. 4c). These observations are important to avoid the misinterpretation of isomeric ions originating from in-source fragmentation.

#### Classification of unknowns and differentiation between isomers

In addition to CBE and the CBE isomer described above, several unknown peaks were observed in the spectra of CBD e-liquid formulations throughout 4 weeks of storage at 40 °C and 75% RH, some of which also had the same mass as CBE. As shown in Fig. 3a and b, the HRXIC of the *m/z* 331.2259 ion contained several peaks corresponding to isomeric compounds. The same observation was made when the *m/z* 347.2217 and *m/z* 329.2170 ions were extracted (Supplementary Fig. S3 and S7). These isomeric unknown compounds were classified based on their MS^2^ fragmentation and the presence of diagnostic fragment ions.

In the MS^2^ fragmentation pattern of CBD, the main fragment ion at *m/z* 193.1228 with an elemental composition of C_12_H_17_O_2_ (Δppm,− 2.8) corresponds to the olivetol moiety (Fig. 4a). This fragment ion was used as a basis to classify the unknown peaks. For example, unknown compounds with *m/z* 331.2274 (C_21_H_31_O_3_) and *m/z* 347.2220 (C_21_H_31_O_4_) ions, differing from CBD (C_21_H_31_O_2_) by one and two oxygens, respectively, were observed (Fig. 4b and c), suggesting that oxidation had taken place, while the presence of the diagnostic ion *m/z* 193.1228 allowed attribution of an olivetol core structure to these unknown compounds. Thus, the unknown compounds were putatively identified as OH-CBD and diOH-CBD, respectively. Their observed elution from the nonpolar chromatography column, at 3.2 min and 1.9 min, respectively, was consistent with this assignment, because hydroxylation of a compound increases its polarity and leads to earlier elution. The commercially available OH-CBD standards, 6α-OH-CBD and 7-OH-CBD, did not elute at either of these retention times and also showed a different MS^2^ fragmentation pattern; thus, the exact structure of the OH-CBD and diOH-CBD phytocannabinoids remains tentative.

Diagnostic fragment ions *m/z* 205.1227 and *m/z* 135.0448 (Fig. 4d and e) from CBE suggested presence of a putatively identified hydroxylated product, OH-CBE. The earlier elution of the tentatively identified OH-CBE (2.7 min) relative to CBE (4.2 min) is again consistent with an increase in polarity due to hydroxylation.

Overall, the classification of unknown degradation products and differentiation between isomeric compounds, based on the diagnostic fragment ions present in MS^2^ spectra, indicated that the main degradation products in CBD e-liquid samples were related to CBD but additional peaks associated with the hydroxylation of CBE were also observed.

### Trends in CBD stability in e-liquid stored under different conditions

The compounds associated with degradation of CBD and CBE were monitored at weekly intervals in e-liquids stored for 29 days at different conditions of light exposure and temperature (Table 2). The abundances of the degradation products differed with storage condition. For samples kept at 4 °C in the dark, minimal degradation was observed after 29 days, while for those stored at ambient temperature in the dark the abundances of degradation products increased slowly over the same period. Higher temperature and exposure to light both resulted in more rapid formation of most of the degradation products and several additional cannabinoid peaks observed after 4 weeks of storage at 40 °C and 75% RH (Table 3).

**Table 2:** Storage conditions of e-liquid formulations.

Glass vial	Temperature (°C)/humidity (% RH)	Dark / light exposure
Clear	25 °C/60% RH—ambient	Light
Amber	25 °C/60% RH—ambient	Dark
Amber	40 °C/75% RH—stressed	Dark
Amber	4 °C—refrigerated	Dark

**Table 3:** Cannabinoids, impurities, and degradation products detected in CBD e-liquids stored for 4 weeks under stress conditions (40 °C/75% RH).

RT (min)	Cannabinoid	*m/z*	Adduct	Elemental composition	Mass accuracy (Δppm)	ΔCCS_TIMS_ (%)^a^	Synthetic CBD	Isolate CBD	Validated^b^
1.84	6αOH-CBD	331.2265	[M + H]^+^	C_21_H_31_O_3_	0.7	0.1	No	Yes	Yes
2.03	7-OH CBD	331.2266	[M + H]^+^	C_21_H_31_O_3_	0.6	0.6	No	Yes	Yes
2.13	diOH-CBD^c^	347.2216	[M + H]^+^	C_21_H_31_O_4_	1.42		Yes	Yes	No
2.42	HU-331-like^c^	329.2170	[M + H]^+^	C_21_H_29_O_3_	1.11	0.3	Yes	No	No
2.52	diOH-CBD^c^	347.2216	[M + H]^+^	C_21_H_31_O_4_	1.39		Yes	Yes	No
2.76	OH-CBE^c^	347.2216	[M + H]^+^	C_21_H_31_O_4_	− 0.12		Yes	Yes	No
2.85	CBE isomer^c^	331.2263	[M + H]^+^	C_21_H_31_O_3_	1.11	0.3	Yes	Yes	No
3.20	CBDV	287.2003	[M + H]^+^	C_19_H_27_O_2_	0.76	0.69	No	Yes	Yes
3.22	OH-CBD^c^	331.2257	[M + H]^+^	C_21_H_31_O_3_	2.42		Yes	Yes	No
3.61	OH-CBE^c^	347.2216	[M + H]^+^	C_21_H_31_O_4_	1.68		Yes	Yes	No
3.67	OH-CBD^c^	331.2261	[M + H]^+^	C_21_H_31_O_3_	1.38		Yes	No	No
3.92	CBDB	301.2160	[M + H]^+^	C_20_H_29_O_2_	0.48	0.6	Yes	Yes	Yes
4.17	CBE	331.2261	[M + H]^+^	C_21_H_31_O_3_	− 1.27	0.6	Yes	Yes	Yes
4.21	OH-CBE^c^	347.2213	[M + H]^+^	C_21_H_31_O_4_	1.03		Yes	Yes	No
4.75	OH-CBE^c^	347.2216	[M + H]^+^	C_21_H_31_O_4_	0.2	N/A	Yes	Yes	No
4.99	CBD	315.2319	[M + H]^+^	C_21_H_31_O_2_	− 0.01	0.2	Yes	Yes	Yes
6.56	CBDH	329.2474	[M + H]^+^	C_22_H_33_O_2_	0.33	0.2	No	Yes	Yes
8.95	CBDP	343.2632	[M + H]^+^	C_23_H_35_O_2_	0.11	0.6	No	Yes	Yes
9.65	CBDH isomer^c^	329.2472	[M + H]^+^	C_22_H_33_O_2_	0.89		Yes	No	No
9.92	HU-331	329.2109	[M + H]^+^	C_21_H_29_O_3_	− 0.46	0.3	Yes	Yes	Yes

*CBD* cannabidiol; *CBDB* cannabidibutol; *CBDH* cannabidihexol; *CBDP* cannabidiphorol; *CBDV* cannabidivarin; CBD-HQ, *CBD* hydroxyquinone (HU-331); *CBE* cannabielsoin; *diOH-CBD* dihydroxy-cannabidiol; *OH-CBD* hydroxy cannabidiol; *OH-CBE* hydroxy-cannabielsoin.

^a^ΔCCS (%) values are result of comparison between was estimated by comparing the measured CCS values in analysed samples and CCS values with that obtained using for the certified reference material (CRM) on the same instrument.

^b^Compounds validated by a matching to an authentic standard based on retention time, exact mass measurement, isotopic pattern, collisional cross section, and MS^2^ fragmentation.

^c^Putative identification.

**Figure 5:**
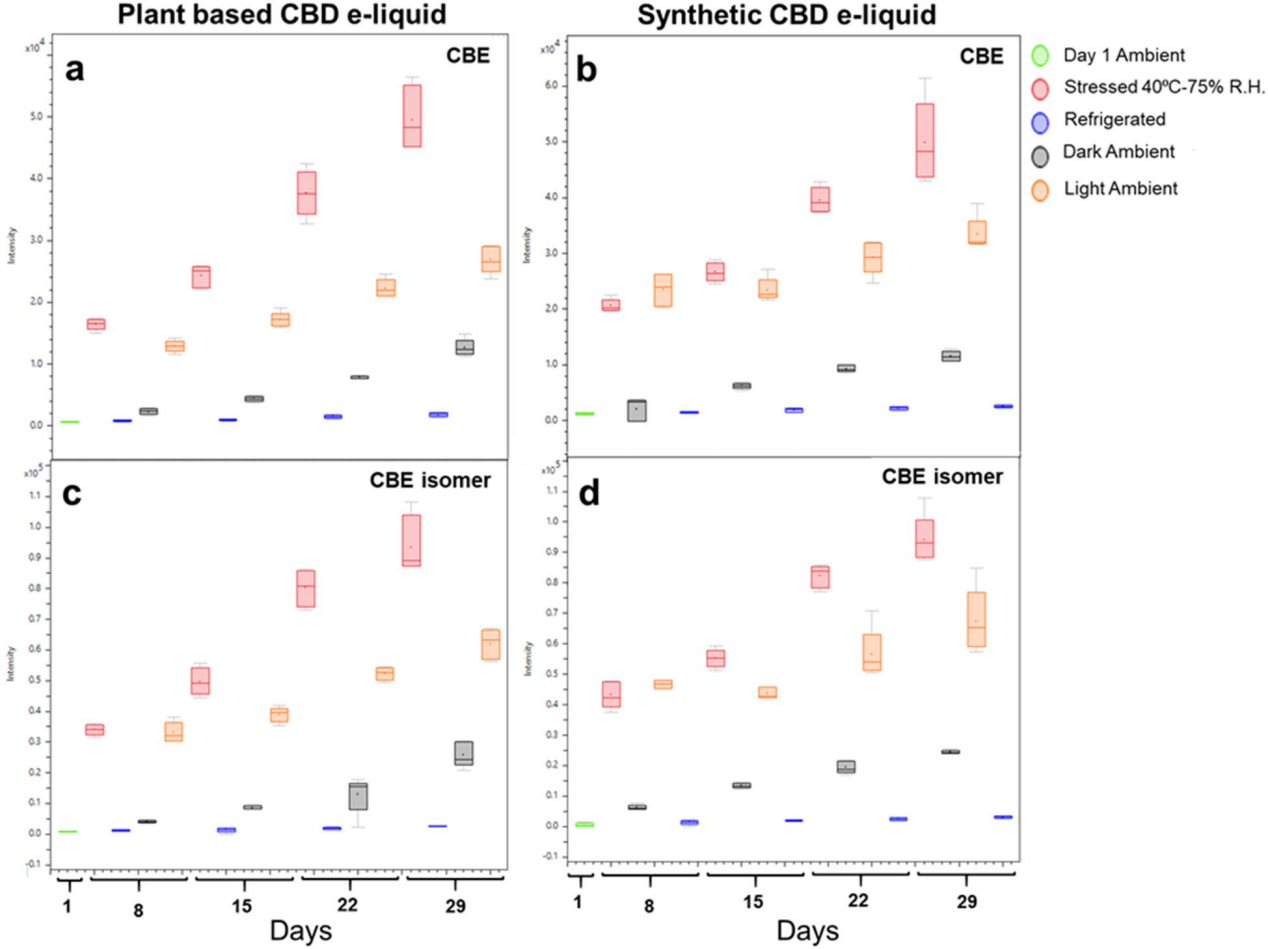
Trends in levels of CBE and CBE isomer in CBD-containing e-liquids over 29 days of storage under four different conditions. (**a**) CBE in plant-based CBD e-liquid; (**b**) CBE in synthetic CBD e-liquid; (**c**) CBE isomer in plant-based CBD e-liquid; and (**d**) CBE isomer in synthetic CBD e-liquid.

CBE and CBE isomer were the most abundant degradation products observed over the study period in e-liquids containing CBD from plant and synthetic sources (Fig. 5). The concentrations were minimal in e-liquid stored at 4 °C, increased in e-liquid stored in the dark at ambient temperature and increased substantially both at 40 °C/75% RH and with light exposure (Fig. 5).

Not all degradation products were highest in abundance under stressed (40 °C/75% RH) conditions. For example, the putatively identified cannabinoid OH-CBE (RT 2.7 min) and its isomer (RT 4.7 min) had greatest abundances in the dark/ambient temperature samples (Supplementary Fig. S8).

The highest concentrations of the CBD oxidation product HU-331 were measured in samples stored at 4 °C in the dark and the lowest concentrations were observed in samples that were exposed to light (Supplementary Fig. S9), indicating a susceptibility to thermal degradation and particularly to photo-degradation.

The profiles of degradation products observed in e-liquids was not affected by the source of CBD (*i.e.* plant or synthetic). The other impurities observed on day 1 of the study (Table 1) remained at similar concentrations for the duration of the study.

## Discussion

Several studies have suggested that CBD may be unstable in some formulations and/or under certain storage conditions^8–10,13^ but there are relatively few published data on the products of CBD degradation, possibly due to analytical constraints that include low-resolution MS techniques and the limited commercial availability of authentic cannabinoid standards. In this study, UHPLC-TIMS-TOF-MS, coupled with an in-house spectral library based on the detailed analysis of 39 phytocannabinoids, was used to detect differences in the cannabinoid profiles of CBD-containing liquids and to identify substances that formed during their storage under varying conditions of temperature and light exposure.

In comparison to synthetic CBD, plant-based CBD isolate in e-liquid formulations was associated with a greater number of impurities. HU-331 was initially detected in e-liquids containing CBD from both sources but the plant-based CBD formulation also contained CBDV, CBDP, CBDH, 6α-OH-CBD and 7-OH-CBD. These plant-specific impurities may act as markers to differentiate CBD from different sources as reported by Citti et al*.*^7,24^. In addition to previously reported diagnostic cannabinoids, CBDP was observed in plant-based CBD in the present study (Table 1 and Fig. 2b). CBDP is a naturally occurring cannabinoid that was isolated from a FM2 cannabis variety for the first time in 2019^24^. To our knowledge, its presence in CBD isolate has not been previously reported. CBDH was also found only in plant-based CBD in the present study and may act as another marker for the identification of plant-based CBD. An unexpected finding was the detection of small amounts of CBDB, a cannabinoid previously reported to be present in plant-based CBD^20^, in the synthetic CBD liquid. To confirm this finding, a second source of synthetic CBD was analysed, and this was also found to contain CBDB. In terms of impurities diagnostic of synthetic CBD, CBDH isomer was putatively identified in both sources of synthetic CBD but not in plant-based CBD.

The instability of CBD in different formulations has been reported^8,9,13^. CBD in sunflower oil decreased in concentration by 20% after 90 days under stress conditions (40 °C, 75% RH, dark), while for CBD in a powder formulation considerably less reduction was observed under the same conditions^8^. CBD in an e-liquid formulation was found to be susceptible to heat and light exposure, with up to 20% reduction occurring after 30 days at 37 °C in the dark and up to 15% reduction at room temperature under natural light exposure^13^. CBD was also found to be unstable under simulated physiological conditions (pH 7.4, 37 °C), with 10% loss observed within 24h^9^. These findings are consistent with the observations of CBD breakdown products in the present study.

To our knowledge, only one previous study has reported the detection of products of CBD breakdown. Among multiple peaks observed in low-resolution LC–MS spectra of partially degraded CBD in sunflower oil^8^, CBN was identified and several unconfirmed oxidation products of CBD were indicated by the presence of [M + H]^+^ ions at *m*/*z* 331.1 and 347.3. CBN was not detected among the degradation products of CBD in the present study but oxidation products were indicated by ions of *m/z* 331.1 and 347.3. The presence of CBE and HU-331 was confirmed in the present study and other phytocannabinoids, including OH-CBE, OH-CBD, diOH-CBD and an HU-331-like derivative, were putatively identified. Psychoactive cannabinoids (*e.g.,* Δ9-THC, Δ8-THC or tetrahydrocannabivarin) were not detected under the conditions applied in the study.

CBE, originally identified as a metabolite of CBD in rat liver^25,26^, and OH-CBD and diOH-CBD cannabinoids, also known metabolites of CBD in mammals^27^, were found to increase in CBD e-liquids under stress conditions (40 °C/75% RH). In contrast, HU-331, a reported oxidation product of CBD^14^, deviated from this trend and was increased in the samples kept at 4 °C and ambient temperature in the dark (Supplementary Fig. S9), indicating the thermal and photo-lability of HU-331. Furthermore, the levels of HU-331 were lowest in samples exposed to natural light, suggesting that HU-331 is most susceptible to photodegradation.

Regarding the mechanisms underlying the breakdown of CBD, oxidation was indicated to be the principal route of degradation, giving rise to hydroxylation and dihydroxylation products. Cyclisation reactions were indicated by the relative abundance of CBE and CBE isomer. Several isomeric degradation products were observed during this study, reflecting the complexity of CBD degradation in e-liquid formulations.

In summary, by constructing a spectral library of 39 reference phytocannabinoid standards and using an untargeted analytical approach at high resolution based on UHPLC-TIMS-TOF-MS, impurities, and degradation products were identified or classified in CBD e-liquid formulations. The results indicated several other impurities that differed between plant-derived and synthetic CBD and which are potentially diagnostic of the CBD source. CBD in e-liquid remained relatively stable when stored at 4 °C in the dark but multiple degradation products, including CBE and CBE stereoisomer, were formed when the e-liquid was stored at ambient and elevated temperatures.

## Methods

### Materials

Acetonitrile Optima LC–MS grade was purchased from Fisher Scientific (Thermo Fisher Scientific, UK) and formic acid LC–MS grade was purchased from Greyhound Ltd (Biosolve, France). Ultra-pure water Milli-Q 18 mΩ was obtained from IQ 7000 Milli-Q system (Millipore, France). ESI-L Low-Concentration Agilent Tuning Mix was obtained from Crawford Scientific (Agilent Technologies, USA). Synthetic CBD material from supplier A and plant-based CBD from supplier C used in this study were > 99% pure and were sourced from independent US-based manufacturers. Single batches of pharmaceutical grade propylene glycol and glycerol were obtained from Sigma-Aldrich (Merk, Germany). The following certified reference materials were purchased from Sigma-Aldrich (Merk, USA): cannabicyclol (CBL), 11-hydroxy-Δ9-tetrahydrocannabinol (11-OH-Δ9-THC), cannabigerolic acid (CBGA), Δ9-tetrahydrocannabinolic acid A (THCA), tetrahydrocannabivarin (THCV), cannabidivarin (CBDV), cannabinol (CBN), cannabigerol (CBG), cannabidivarinic acid (CBDVA), Δ8-tetrahydrocannabinol (Δ8-THC), Δ9-tetrahydrocannabinol (Δ9-THC), cannabicyclolic acid (CBLA), cannabichromenic acid (CBCA), cannabichromene (CBC), cannabidiol (CBD), ( −)-11-nor-9-carboxy-Δ9-THC (11-COOH-Δ9-THC), cannabinolic acid (CBNA), cannabigerolic acid (CBGA), tetrahydrocannabivarinic acid (THCVA), 7-carboxy-cannabidiol (7-COOH-CBD) and cannabidiolic acid (CBDA). The following certified reference materials were purchased from Cambridge Bioscience (Cayman, USA): Cannabicitran (CBT), cannabielsoin (CBE), cannabichromevarin (CBCV), cannabidiol hydroxyquinone (CBD-HQ/HU-331), cannabinodiol (CBND), cannabigerovarinic acid (CBGVA), cannabidiphorol (CBDP), cannabinolmonomethlyether (CBNM), cannabigeroquinone (CBGQ), cannabichromenquinone (CBCQ), 6-α-hydroxy-cannabidiol (6-α-OH-CBD), cannabidibutol (CBDB), cannabichromevarinic acid (CBCVA), cannabichromeorcin (CBCO), cannabidihexol (CBDH), cannabidiol monomethyl ether (CBDM), and Δ9-tetrahydrocannabihexol (Δ9-THCH).

### CBD e-liquid preparation and storage

In brief, CBD material, either plant-based isolate or synthetic, was prepared in PG:VG (70: 30 v/v) in accordance with an internally documented procedure. After preparation, the e-liquids were divided into vials for the different storage conditions. Samples stored under light and ambient temperature were placed in clear vials in a Fitotron SGC 120 (Weisstechnik, Germany) under a light/dark cycle of 16/8 h. Samples stored under dark and ambient temperature were placed in amber vials on a bench in a light-protected sealed bag. Samples stored under the stress condition (40 °C/75% RH) were placed in amber vials and stored in a Memmert cabinet (Schwabach, Germany) protected from light. Samples stored under the low-temperature condition were placed in amber vials and stored in a laboratory refrigerator set at 4 °C. Five independent samples of e-liquid were stored for each condition and aliquots were analysed at 1, 8, 15, 22 and 29 days.

### UHPLC parameters

For UHPLC analysis, 60 mg of e-liquid was accurately weighed into a 25-mL amber volumetric flask, diluted with 25 mL of acetonitrile containing 40 ng/mL of labelled THC (D3-Δ9-THC) as internal standard, and vortexed for a few minutes to create a homogeneous solution. Samples were filtered through a 0.2-µm Millex GV (PVDF) syringe filter (Merk Millipore, Ireland) and transferred to a 2-mL LC–MS vial before analysis on an UHPLC 1290 Infinity system (Agilent Technologies, USA) coupled to a TIMS-TOF Pro mass spectrometer (Bruker Daltonics, Bremen, Germany). For each sample, 5 µl was injected onto a Poroshell EC-C18 column (3.0 mm × 150 mm, 1.9 µm) with a flow rate of 0.5 mL/min, column oven temperature of 30 °C, and autosampler temperature of 10 °C. Mobile phase A was Milli-Q water containing 0.1% formic acid, and mobile phase B was acetonitrile LC–MS grade containing 0.1% formic acid. The program was isocratic elution at 75% B for 24 min, increasing to 100% B at 25 min, hold at 100% B for 3 min, decreasing to 75% B at 29 min, and lastly hold at 75% B for 2 min. The injection needle was washed with acetonitrile (LC–MS grade) before and after injection for 20 s to avoid potential carryover.

### TIMS-TOF-MS parameters

Ionisation was performed with an Apollo II ESI source in positive and negative modes. The parameters were nitrogen drying gas of 10 L/min of, nebuliser pressure of 2.2 Bar, and temperature of 200 °C. Capillary voltage was 4500 V and 3500 V for positive and negative mode, respectively. The following acquisition parameters were used: TIMS ramp time, 100 ms; scan range, *m/z* 50–1200; ion mobility 1/k0, 0.45–1.25 V.s/cm^2^; stepping collision energy for PASEF, 25–50 eV for positive and negative mode. The total cycle of 0.53 s included one full TIMS-HRMS scan and two MS^2^ PASEF scans. The ion charge control was 7.5 million (Mio) and the MS resolution was 60,000 full width at half maximum. *m/z* and ion mobility calibrations were performed before analysis by using a mixture of sodium formate and Agilent ESI-L Low Concentration Tuning Mix in a ratio of 1:2.5. This mixture was infused constantly and 20 µL was injected before each analysis to facilitate an internal calibration after analysis (Supplementary Table S1).

### Data processing

Raw data files were analysed with MetaboScape version 2021b and Compass DataAnalysis 5.3 (Bruker Daltonics, Germany). MetaboScape T-Rex 4D algorithm was used to extract data automatically from the four-dimensional space, namely, *m/z*, retention time, ion mobility (CCS) and intensity. The software also correlated the MS^2^ data acquired with PASEF to each extracted feature. Recalibration of measured masses was performed using sodium formate (see list of adducts in Supplementary Table S1) and the ion mobility data were recalibrated using ESI L-low concentration tuning mix (Agilent Technologies, Germany). Annotation of known cannabinoids was performed using an in-house MS^2^ spectral library containing 39 entries (Supplementary Table S2). MS^2^ spectra of cannabinoids were acquired by analysing mixtures of standards by UHPLC-TIMS-TOF-MS. The resulting spectral MS^2^ library contained exact mass, retention time, isotopic profile, CCS_TIMS_ values and MS^2^ information for 39 phytocannabinoids.

### CBE Isomer Isolation

A flash chromatography system (Pure Chromatography Systems) was used to isolate the CBE isomer. The CBD e-liquid was injected directly into a FlashPure EcoFlex Silica column (300 g). The initial mobile phase composition of 95% hexane—5% ethyl acetate was increased to 100% ethyl acetate over 30 min. The flow rate was set at 110 mL/min and detection was by UV/ELSD. The fractions obtained from the Flash isolation were analysed by UHPLC-timsTOF using the method indicated above. The fraction containing the CBE isomer [M + H]^+^ m/z 331.2263 with the RT 2.85 min was used for structural elucidation by NMR spectroscopy.

### NMR spectroscopy

^1^H-NMR spectra, correlation spectroscopy (COSY), selective total correlation spectroscopy (sel. TOCSY), nuclear Overhauser effect spectroscopy (NOESY), heteronuclear single quantum coherence (HSQC) and heteronuclear multi bond correlation (HMBC) spectra were obtained using a Bruker Advance 600 MHz NMR spectrometer with a 5 mm broadband inverse probe. Chemical shifts (δ) are in part per million (ppm), coupling constant are reported in hertz (Hz). Splitting patterns are represented as follow: singlet (s), doublet (d), triplet (t), quartet (q), double doublet (dd), quintet (qnt), multiplet (m), and broad singlet (bs).

^1^H NMR (600 Hz, CD_3_CN), δ 6.25 (s, 1H), 6.24 (s, 1H), 6.16 (s, 1H), 4.72 (m, 1H), 4.64 (m, 1H), 4.12 (d, *j* = 5.43 Hz, 1H), 3.23 (dd, *j* = 10.90 Hz, 5.43 Hz, 1H), 2.89 (bs, 1H), 2.47 (t, *j* = 7.73 Hz, 2H), 1.91 (m, 1H), 1.81–1.73 (m, 4H), 1.65 (m, 1H), 1.59–1.48 (m, 4H), 1.37–1.23 (m, 7H), 0.89 (t, *j* = 7.12 Hz, 3H).

## Supplementary Information

Supplementary Information.
