# Supplementary material for: Characterizing the degradation of cannabidiol in an e-liquid formulation
Source: Sci Rep. 2022 Nov 21;12:20058. doi: 10.1038/s41598-022-23910-6 (PMC9681878; doi:10.1038/s41598-022-23910-6)
Supplement: Supplementary file 1 — Supplementary Information. [file 41598_2022_23910_MOESM1_ESM.pdf]

Supporting Information

Characterizing the degradation of cannabidiol in an e-liquid formulation

Adrián Schwarzenberg[1], Harry Carpenter[1], Christopher Wright[1], Omer Bayazeid[1], and Michał Brokl[1*]

[1]B.A.T. (Investments) Limited, Southampton SO15 8TL, United Kingdom

*Corresponding author: michal_brokl@bat.com

Supplementary tables

Table S1. List of sodium formate adducts used for mass calibration.

Mass Calibration | Mass Calibration Graph

Calibration spectrum: 4.2-9.7s

Calibration mode: hpc8

Standard deviation: 0.04 mDa

0.06 ppm

Reference ...	Resulting m...	Intensity	Error [ppm]	Error [mDa]
90.97665	90.97665	801389	0.00	0.00
158.96407	158.96407	221493	-0.01	-0.00
226.95149	226.95150	685967	0.04	0.01
294.93892	294.93891	37350	-0.03	-0.01
362.92634	362.92633	246528	-0.04	-0.01
430.91377	430.91381	373087	0.10	0.04
498.90119	498.90116	246828	-0.05	-0.03
566.88861	566.88859	250970	-0.04	-0.02
634.87604	634.87606	235991	0.03	0.02
702.86346	702.86348	251574	0.03	0.02
770.85088	770.85087	206959	-0.01	-0.01
838.83831	838.83827	161352	-0.05	-0.04
906.82573	906.82579	91843	0.07	0.06
974.81316	974.81312	55441	-0.04	-0.04
1042.80058	1042.80059	31191	0.01	0.01
1110.78800	1110.78800	16649	-0.00	-0.00

Table S2. The 39 standards used to build the mass spectral library.

Standard	Molecular weight	Formula	CAS no.
CBCV	286.19	$C_{19}H_{26}O_2$	41408-19-9
CBN	310.19	$C_{21}H_{26}O_2$	521-35-7
Δ8-THC	314.22	$C_{21}H_{30}O_2$	5957-75-5
CBG	316.24	$C_{21}H_{32}O_2$	25654-31-3
CBNM	324.21	$C_{22}H_{28}O_2$	41935-92-6
CBD-HQ	328.2	$C_{21}H_{28}O_3$	137252-25-5
CBDVA	330.18	$C_{20}H_{26}O_4$	31932-13-5
CBGVA	332.2	$C_{20}H_{28}O_4$	64924-07-8
CBDP	342.26	$C_{23}H_{34}O_2$	55824-13-0
(−)-11-nor-9-Carboxy-Δ9-THC	344.2	$C_{21}H_{28}O_4$	56354-06-4
CBNA	354.18	$C_{22}H_{26}O_4$	2808-39-1
THCA	358.21	$C_{22}H_{30}O_4$	23978-85-0
CBGA	360.23	$C_{22}H_{32}O_4$	25555-57-1
CBGQ	330.22	$C_{21}H_{30}O_3$	1884127-66-5
THCV	286.19	$C_{19}H_{26}O_2$	31262-37-0
CBND	310.19	$C_{21}H_{26}O_2$	39624-81-2
Δ9-THC	314.22	$C_{21}H_{30}O_2$	1972-08-3
CBCQ	328.2	$C_{21}H_{28}O_3$	N/A
THCVA	330.18	$C_{20}H_{26}O_4$	39986-26-0
CBE	330.22	$C_{21}H_{30}O_3$	52025-76-0
CBLA	358.21	$C_{22}H_{30}O_4$	40524-99-0
CBDV	286.19	$C_{19}H_{26}O_2$	24274-48-4
CBD	314.22	$C_{21}H_{30}O_2$	13956-29-1
6α-hydroxy-CBD	330.22	$C_{21}H_{30}O_3$	58940-28-6
CBCA	358.21	$C_{22}H_{30}O_4$	185505-15-1
CBDB	300.44	$C_{20}H_{28}O_2$	60113-11-3
CBT (citran)	314.2	$C_{21}H_{30}O_2$	31508-71-1
7-Carboxy-CBD	344.44	$C_{21}H_{28}O_4$	1101886-13-8
CBCVA	330.18	$C_{20}H_{26}O_4$	1628112-69-5
CBDA	358.21	$C_{22}H_{30}O_4$	1244-58-2
7-Hydroxy-CBD	330.22	$C_{21}H_{30}O_3$	1101886-10-5
CBCO	258.4	$C_{17}H_{22}O_2$	55824-09-4
11-Hydroxy-THC	330.22	$C_{21}H_{30}O_3$	34675-49-5
CBC	314.2	$C_{21}H_{30}O_2$	20675-51-8
CBL	314.2	$C_{21}H_{30}O_2$	21366-63-2
CBDM	328.5	$C_{22}H_{32}O_2$	1972-05-0
CBCB	300.4	$C_{20}H_{28}O_2$	2552823-92-2
CBDH	328.5	$C_{22}H_{32}O_2$	2552798-21-5
THCH	328.5	$C_{22}H_{32}O_2$	36482-24-3

Supplementary figures

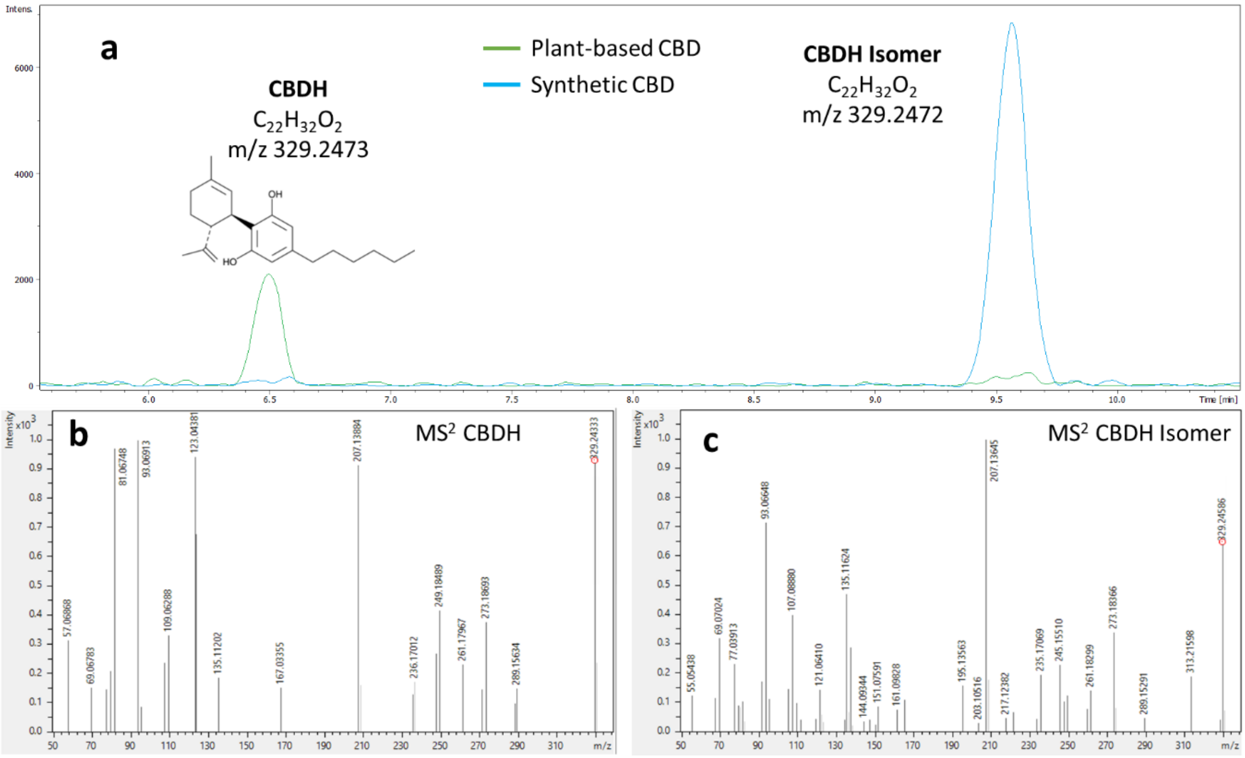

Figure S1. CBD source-specific impurities. **a**) High-resolution extraction ion chromatogram (HRXIC) of CBDH found only in plant-based CBD (green trace) and that of CBDH isomer found only in synthetic CBD (blue trace). MS^2 fragmentation pattern of CBDH in plant-based CBD e-liquid (**b**) and CBDM in synthetic CBD e-liquid (**c**).

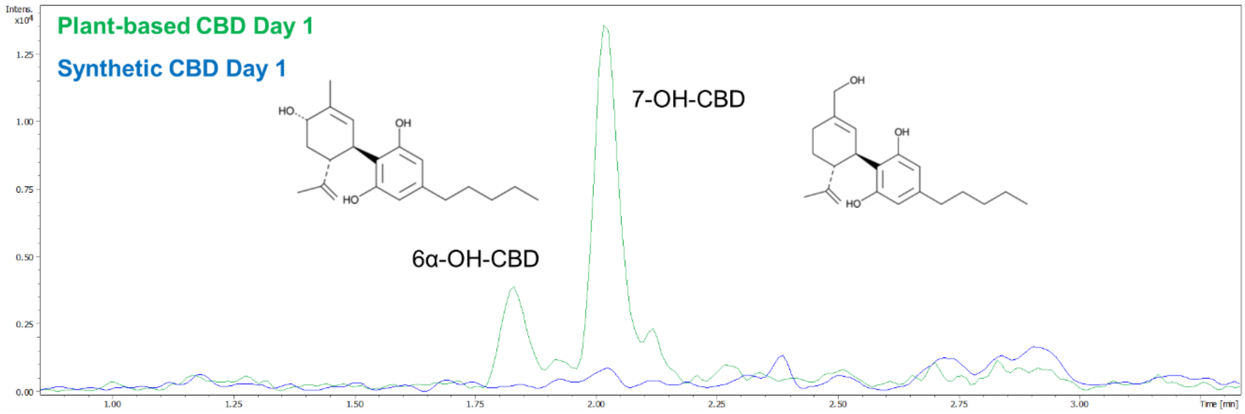

Figure S2. 6-αhydroxy-cannabidiol (6α-OH-CBD) and 7-hydroxy-cannabidiol (7-OH-CBD) identified only in plant-based CBD e-liquid.

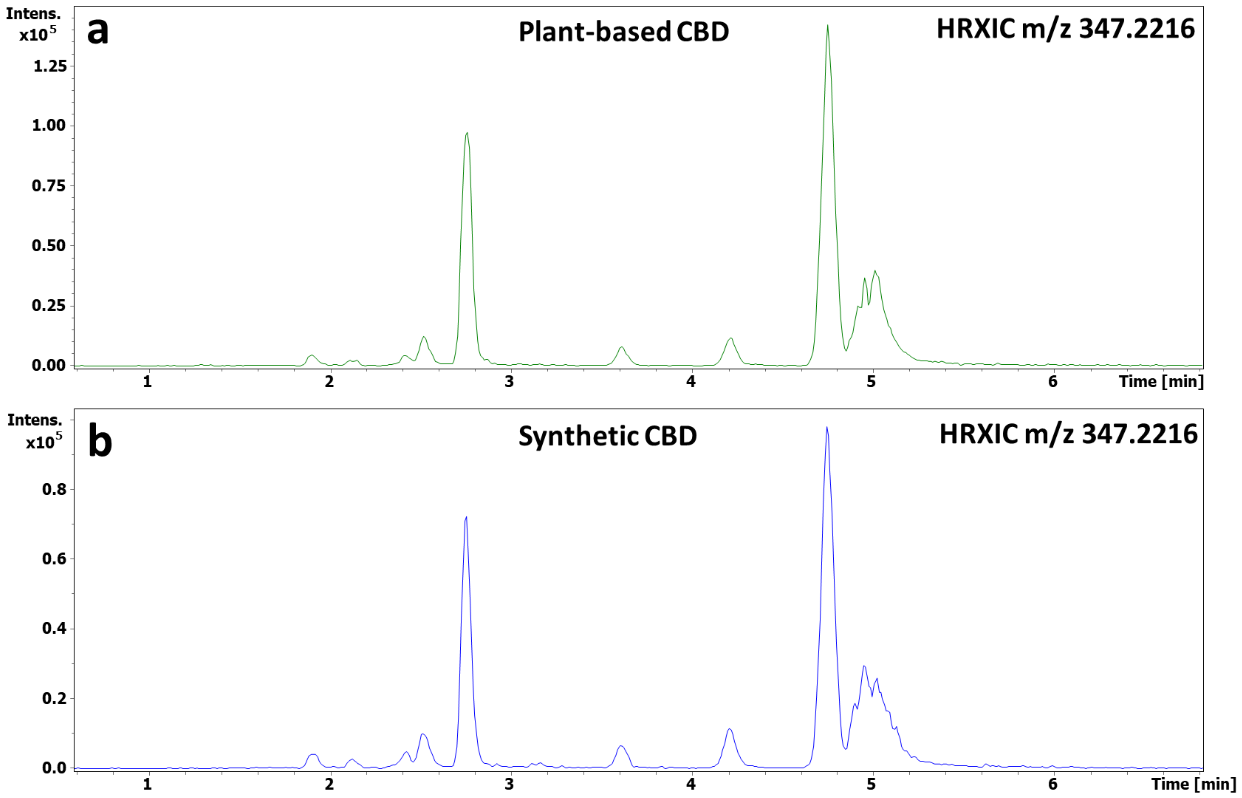

Figure S3. HRXIC of m/z 347.2216 ion observed in CBD e-liquid stored for 29 days under stressed conditions. **a**) Plant-based CBD e-liquid formulation; **b**) synthetic CBD e-liquid formulation.

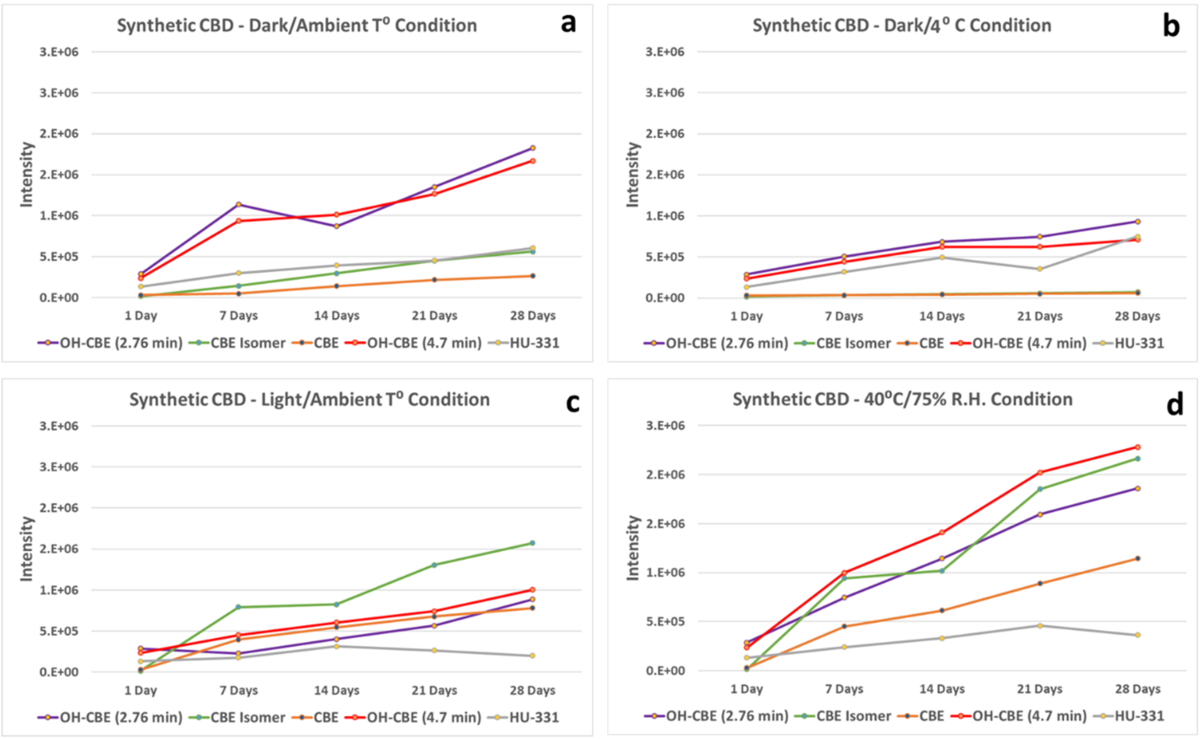

Figure S4. Trends in levels of cannabinoids detected in e-liquid formulated with synthetic CBD stored for 29 days under four different conditions. **a)** Ambient dark storage; **b)** 4 °C dark storage; **c)** ambient light storage; **d)** 40 °C/75%RH storage.

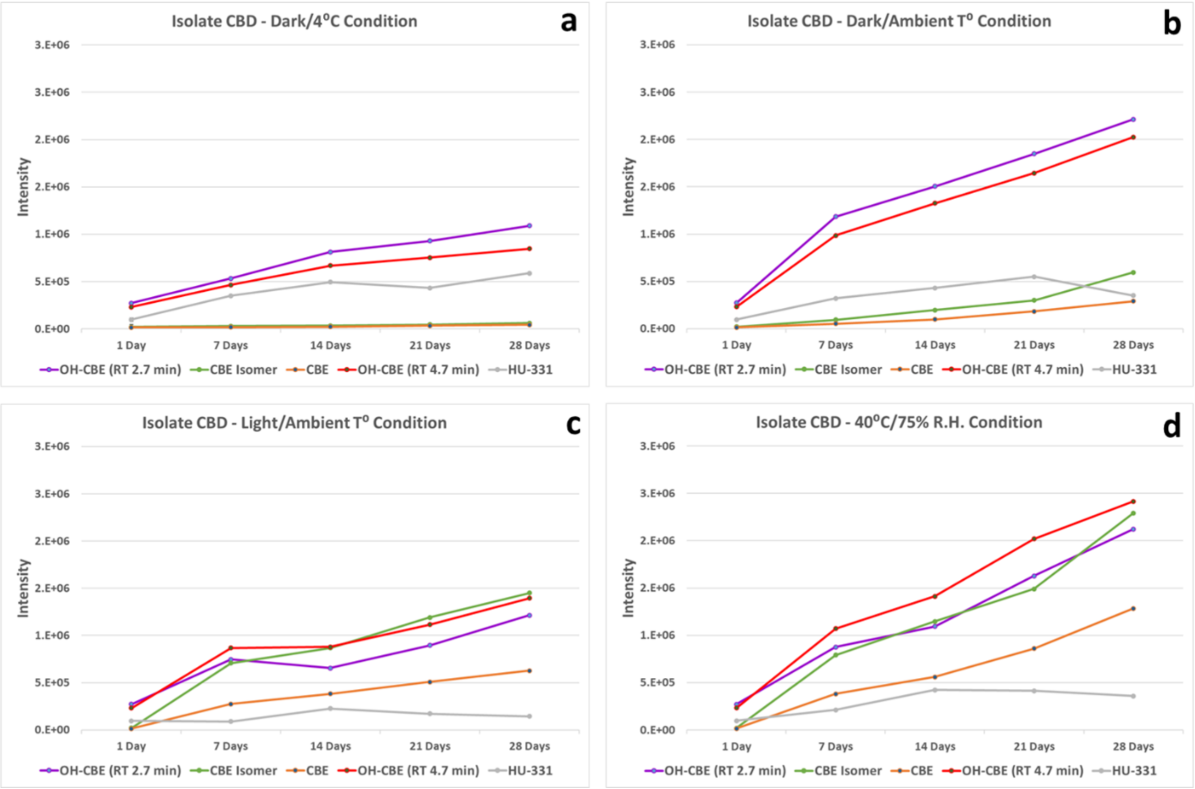

Figure S5. Trends in levels of cannabinoids detected in e-liquid formulated with plant-based CBD stored for 29 days under four different conditions. **a**) Ambient dark storage; **b)** 4 °C dark storage; **c**) ambient light storage; **d)** 40 °C/75%RH storage.

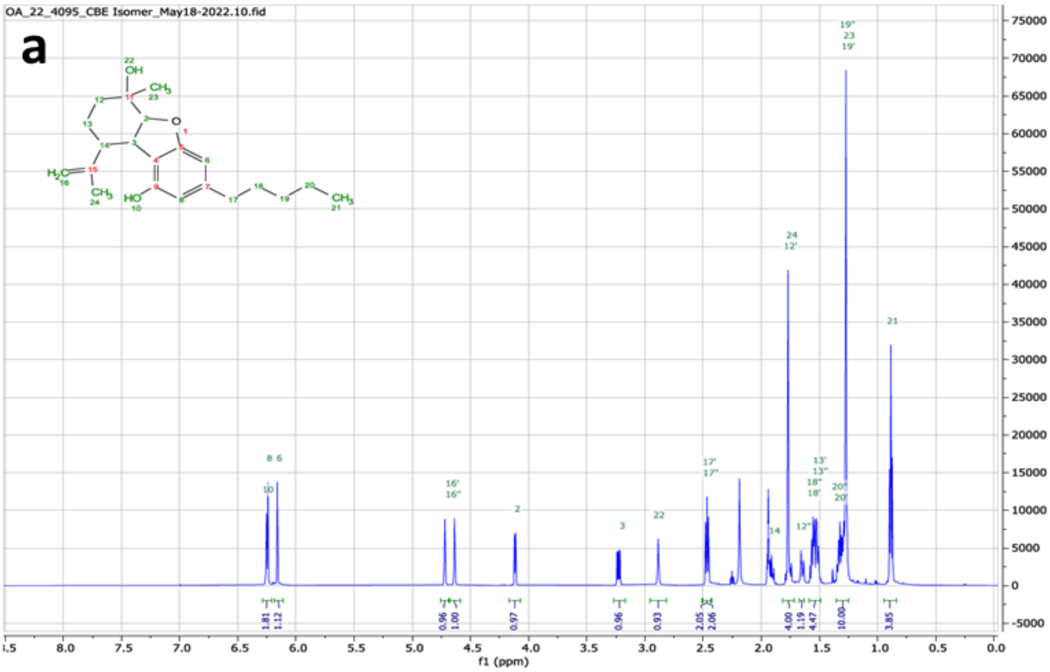

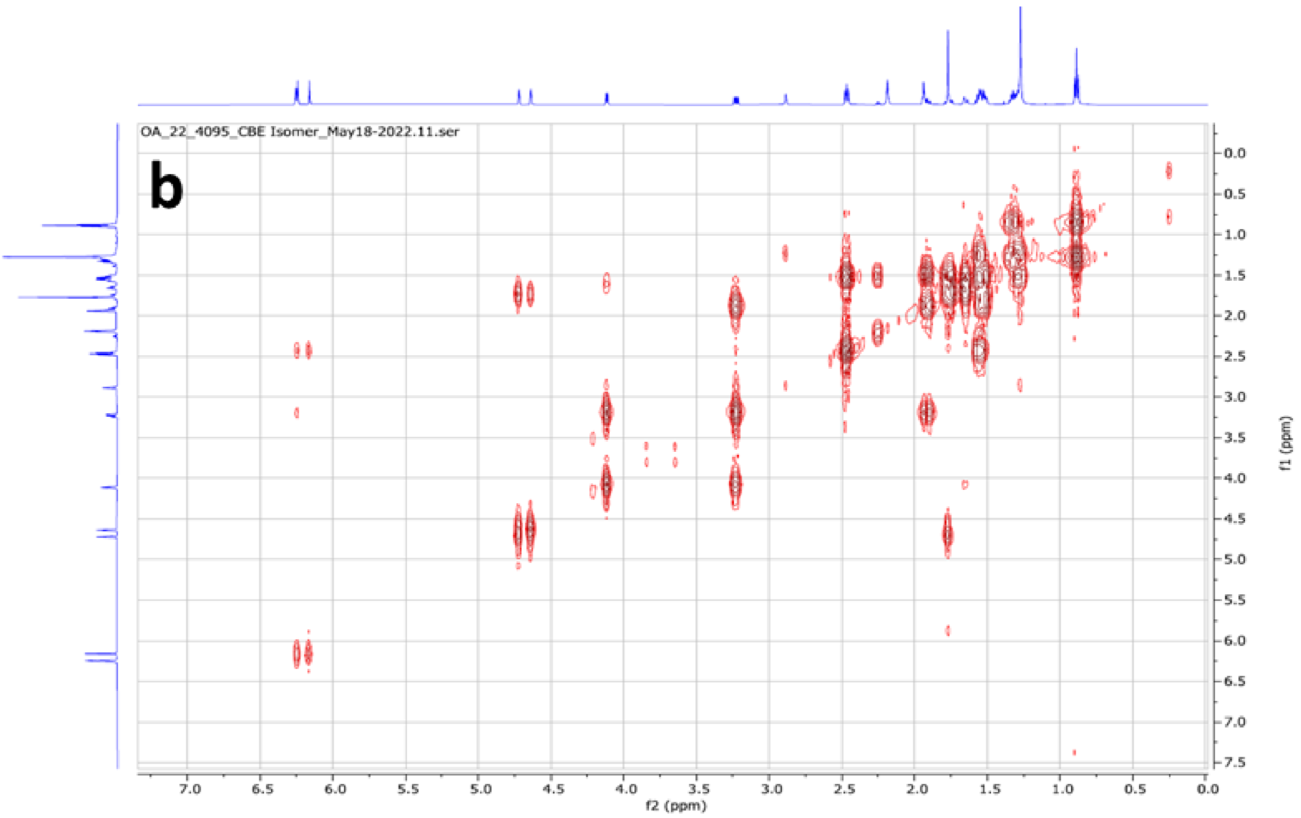
OA_22_4095_CBE Isomer_May18-2022.11.ser
b
f2 (ppm)
f1 (ppm)

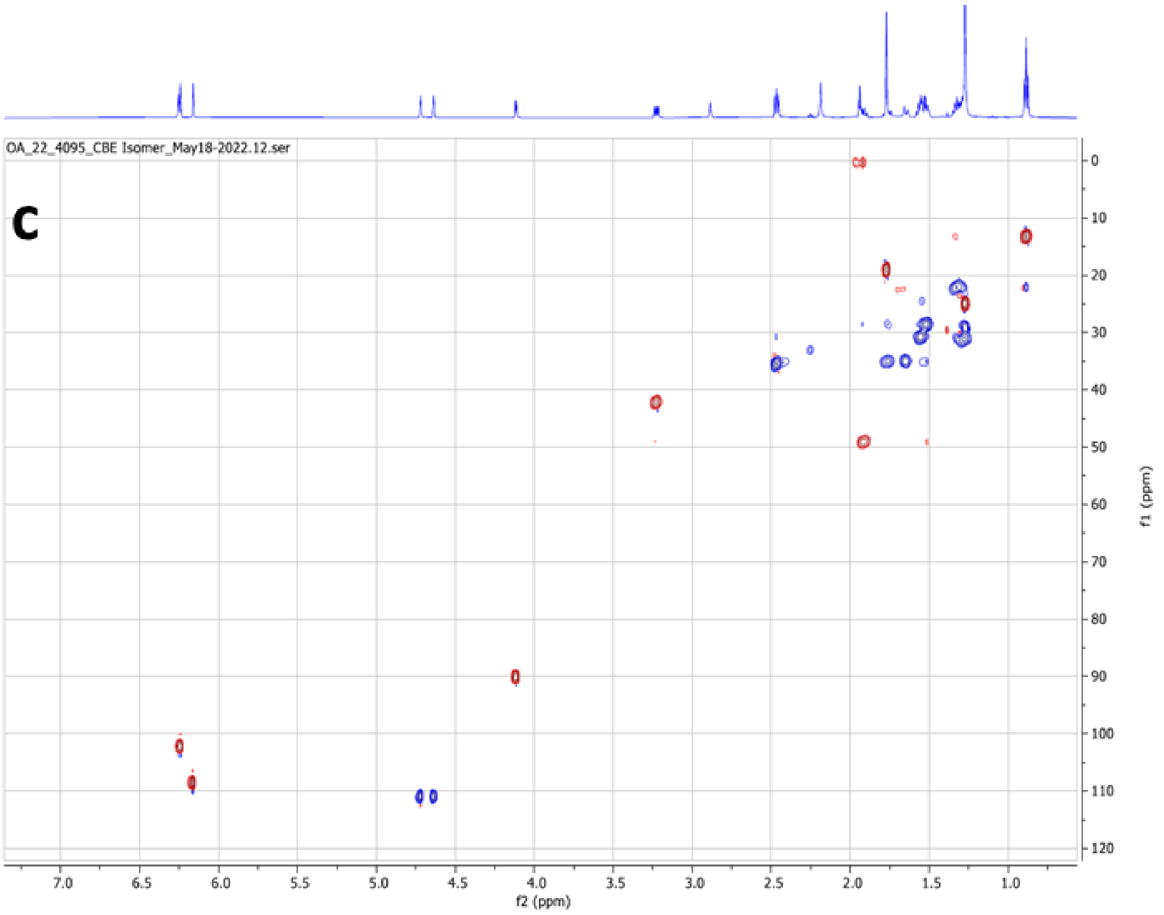
OA_22_4095_CBE Isomer_May18-2022.12.ser
c
f2 (ppm)
f1 (ppm)

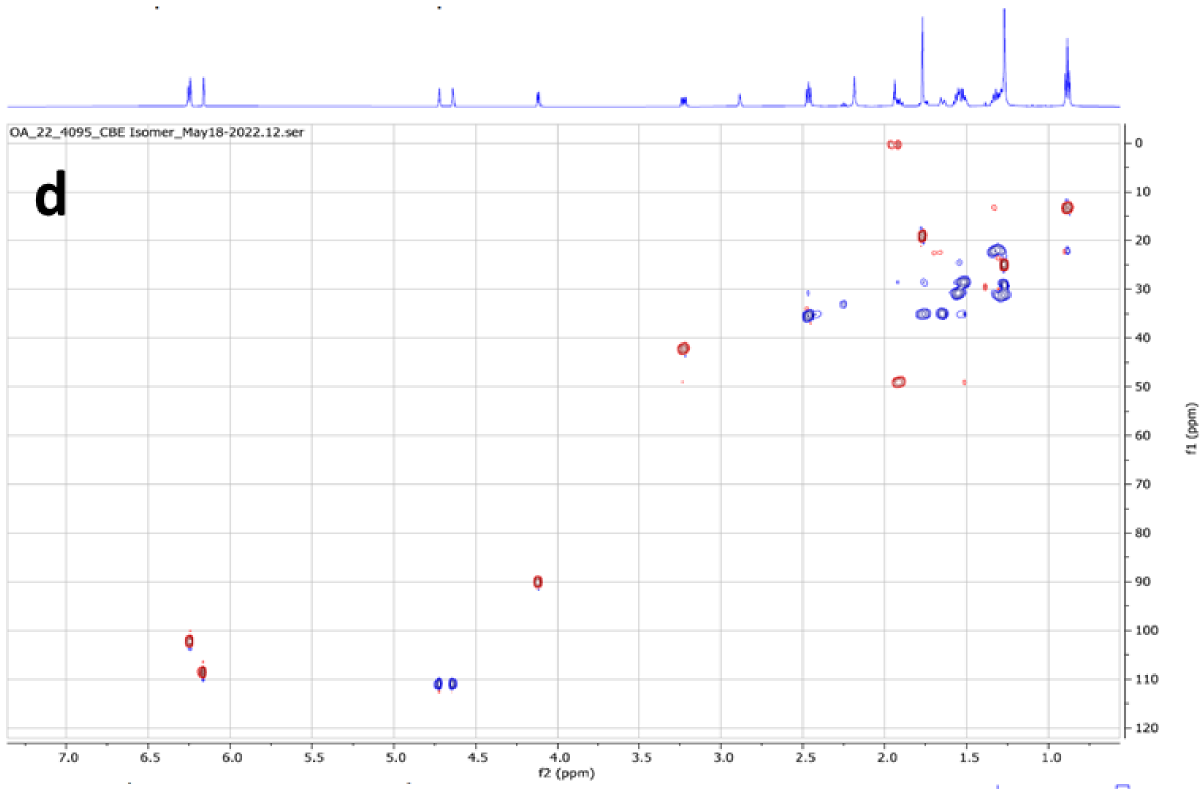

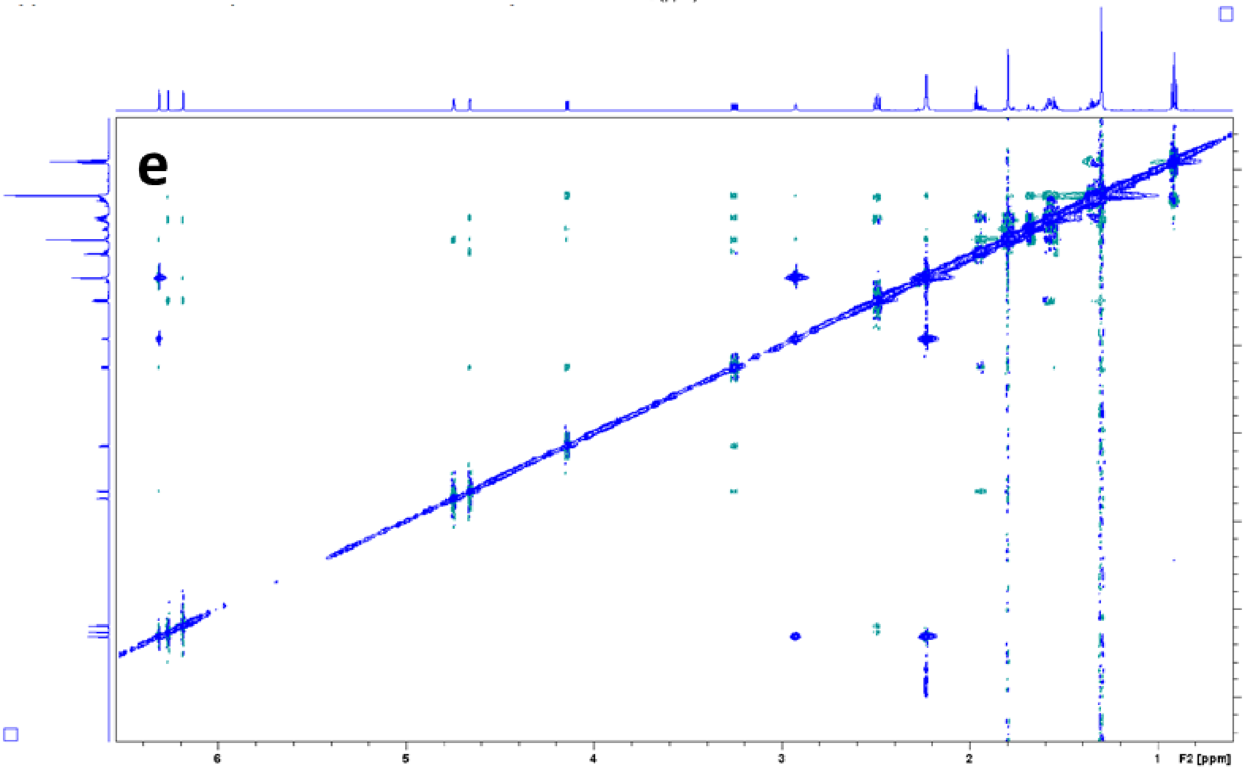

Figure S6. NMR analysis of CBE isomer, **a)** ^{1}H-NMR spectrum in CD_3CN, **b)** COSY spectrum in CD_3CN, **c)** HSQC spectrum in CD_3CN, **d)** HMBC spectrum of in CD_3CN, and **e)** NOESY spectrum in in CD_3CN.

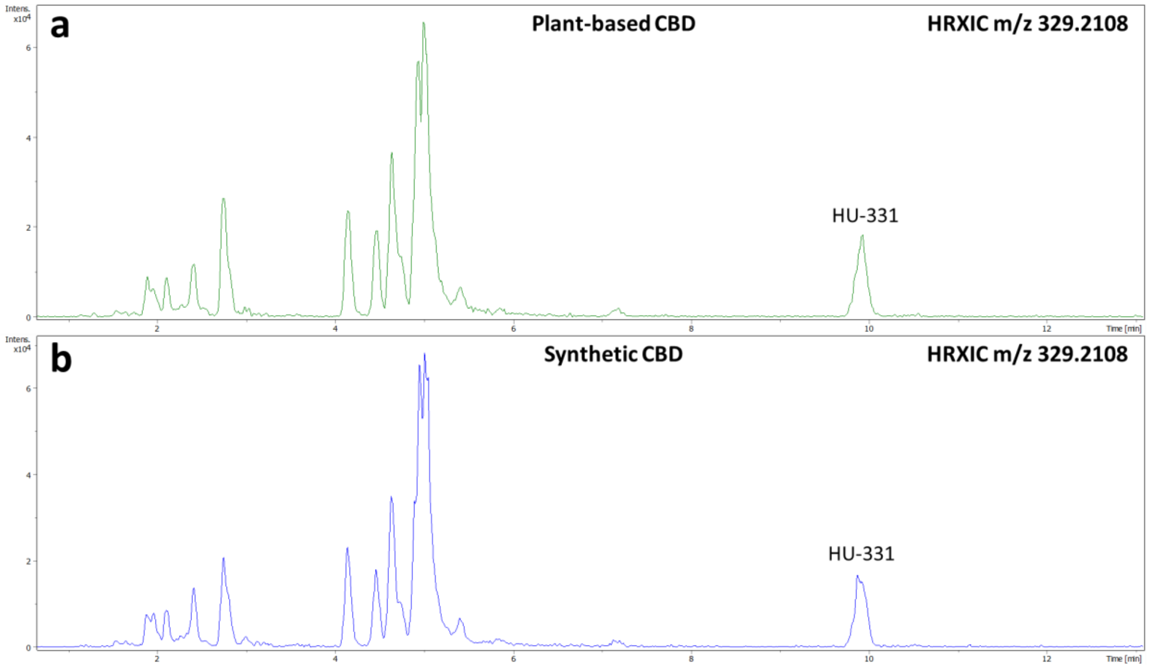

Figure S7. HRXIC of m/z 329.2108 ion observed in CBD e-liquid stored for 29 days under stressed conditions. **a)** Plant-based CBD e-liquid formulation; **b)** synthetic CBD e-liquid formulation.

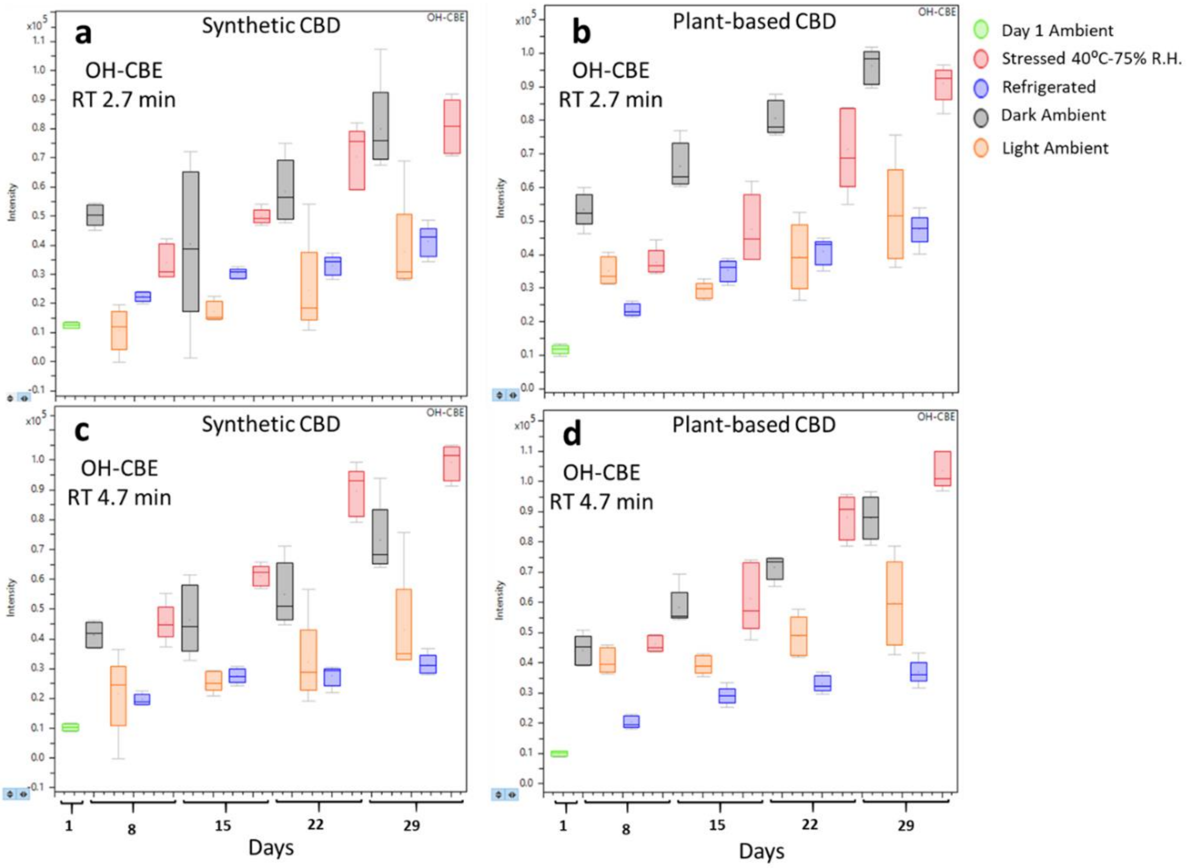

Figure S8. Trend in levels of the putative OH-CBE in CBD e-liquid stored for 29 days under four different conditions. **a**) OH-CBE, RT 2.7 min, synthetic CBD e-liquid formulation; **b**) OH-CBE, RT 2.7 min, plant-based CBD e-liquid formulation; **c**) OH-CBE, RT 4.7 min, synthetic CBD e-liquid formulation; **d**) OH-CBE, RT 4.7 min, plant-based CBD e-liquid formulation.

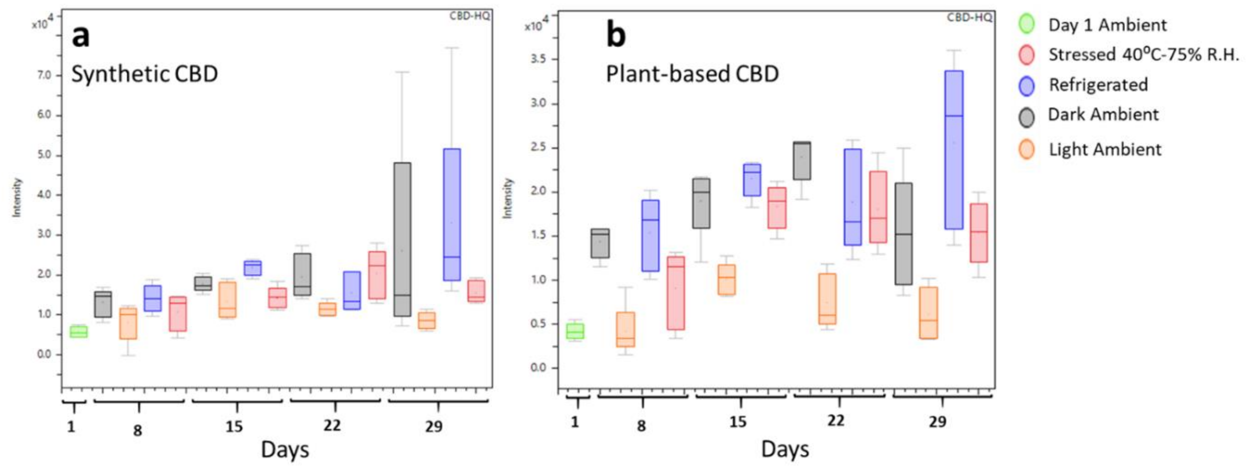

Figure S9. Trend in levels of HU-331 in CBD e-liquid stored for 29 days under four different conditions. **a**) HU-331 in synthetic CBD e-liquid formulation; **b**) HU-331 in plant-based CBD e-liquid formulation.

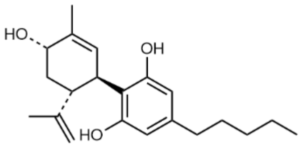

6α–OH-CBD

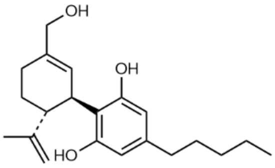

7–OH-CBD

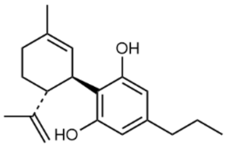

CBDV

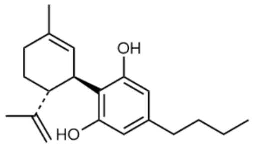

CBDB

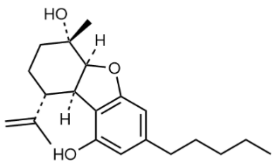

CBE

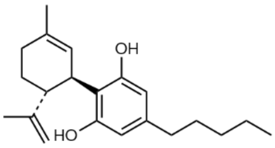

CBD

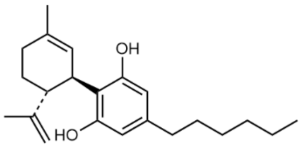

CBDH

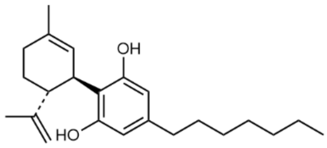

CBDP

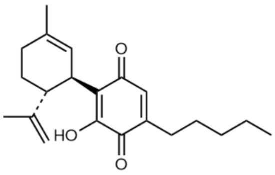

HU-331

Figure S10. Chemical structures of validated cannabinoids.

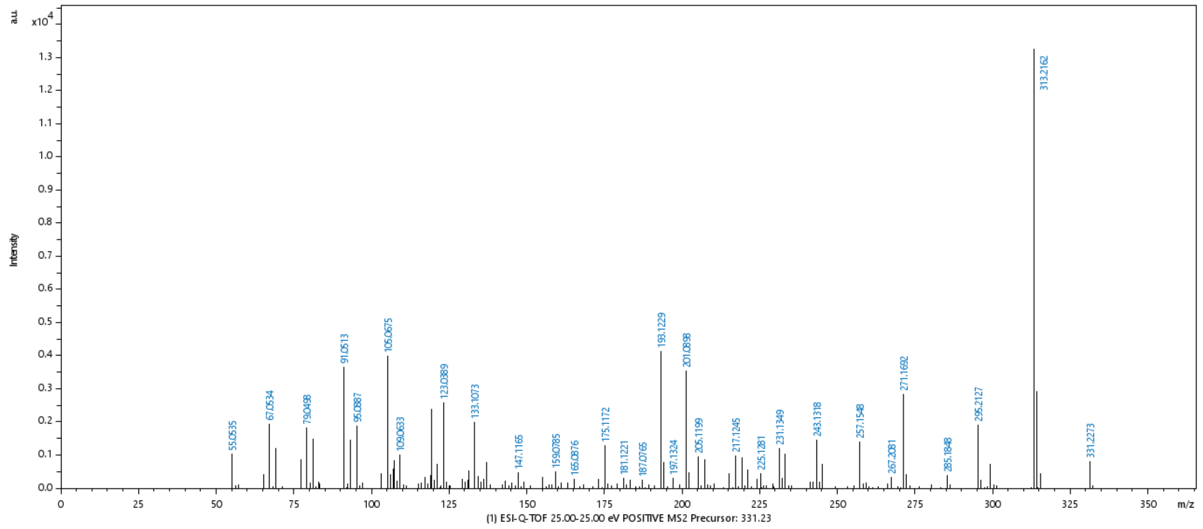

Figure S11. Experimental MS^2 fragmentation pattern of 6α-OH-CBD standard.

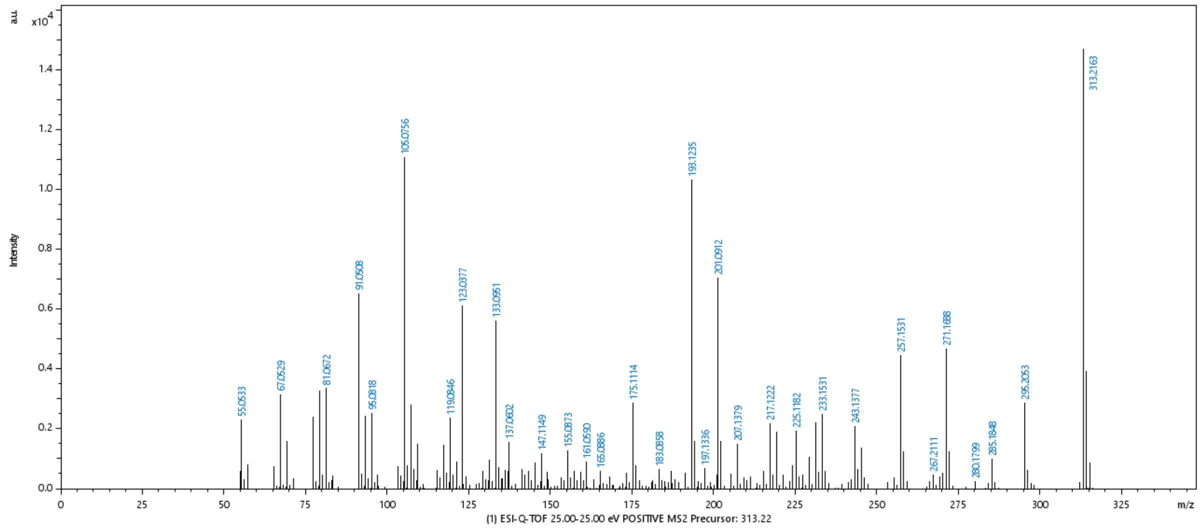

Figure S12. Experimental MS^2 fragmentation pattern of 7-OH-CBD standard.

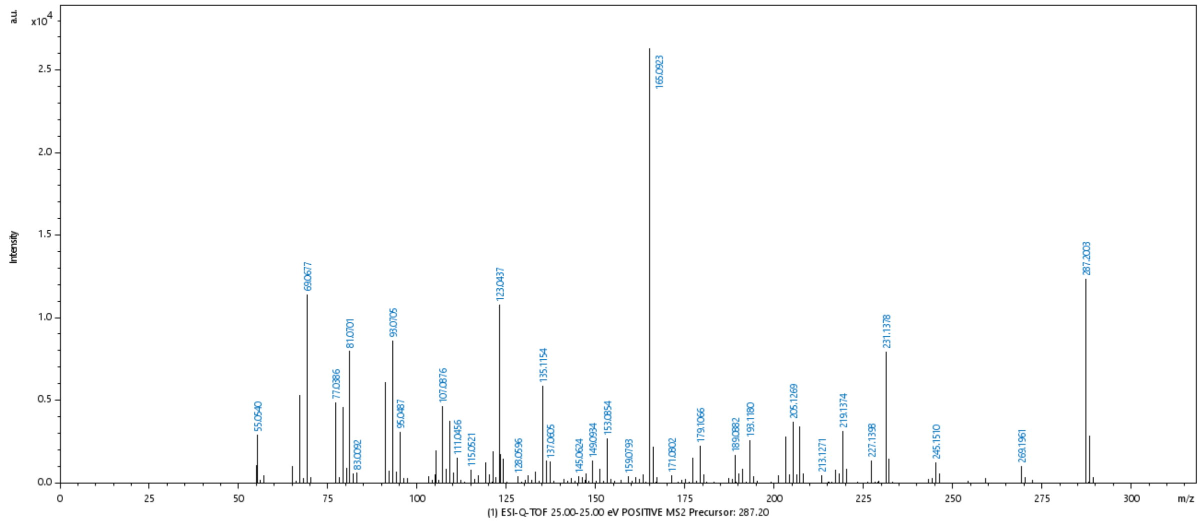

Figure S13. Experimental MS^2 fragmentation pattern of CBDV standard.

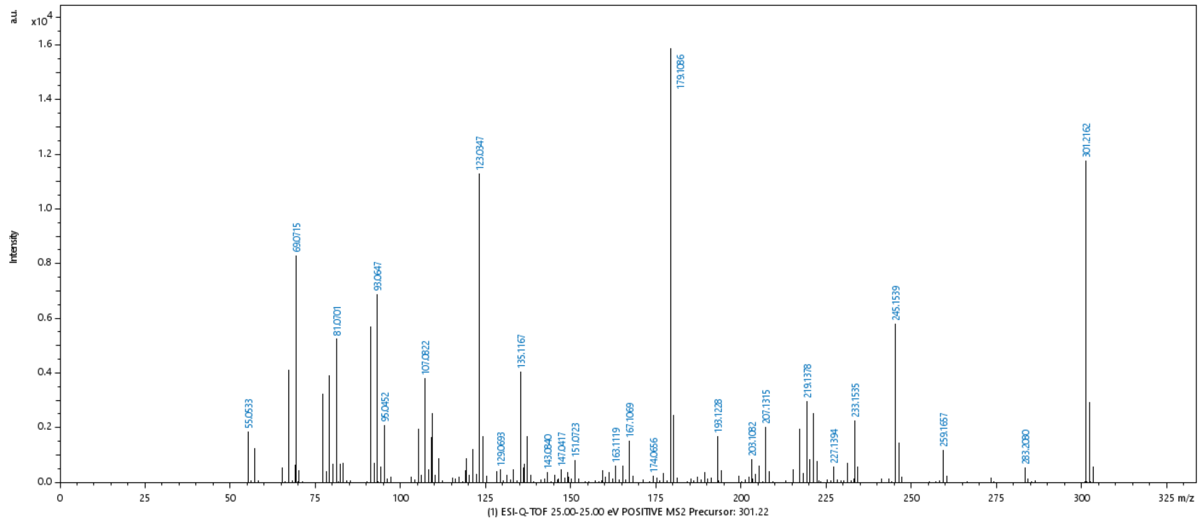

Figure S14. Experimental MS^2 fragmentation pattern of CBDB standard.

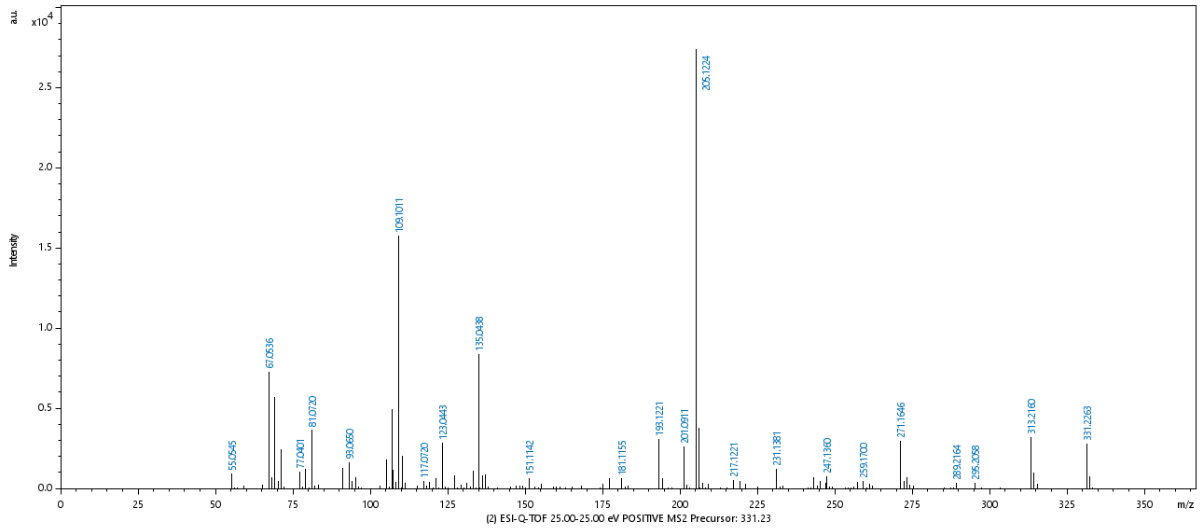

Figure S15. Experimental MS^2 fragmentation pattern of CBE standard.

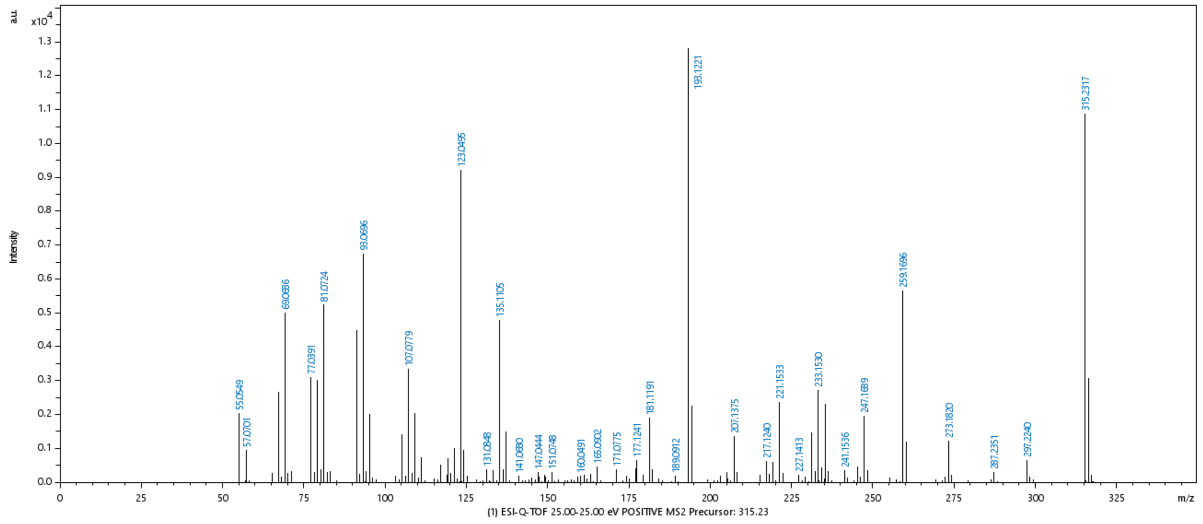

Figure S16. Experimental MS^2 fragmentation pattern of CBD standard.

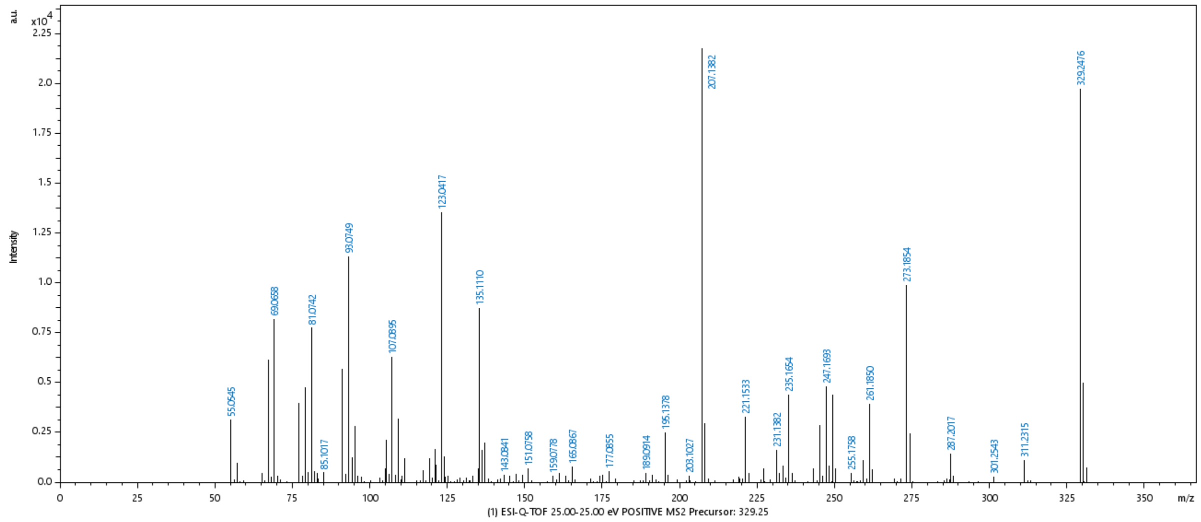

Figure S17. Experimental MS^2 fragmentation pattern of CBDH standard.

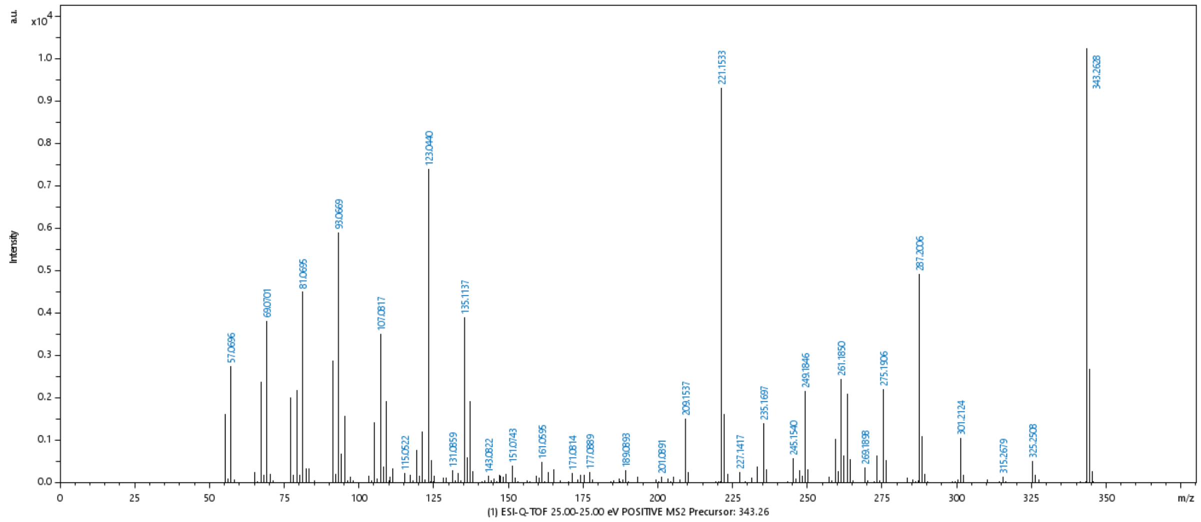

Figure S18. Experimental MS^2 fragmentation pattern of CBDP standard.

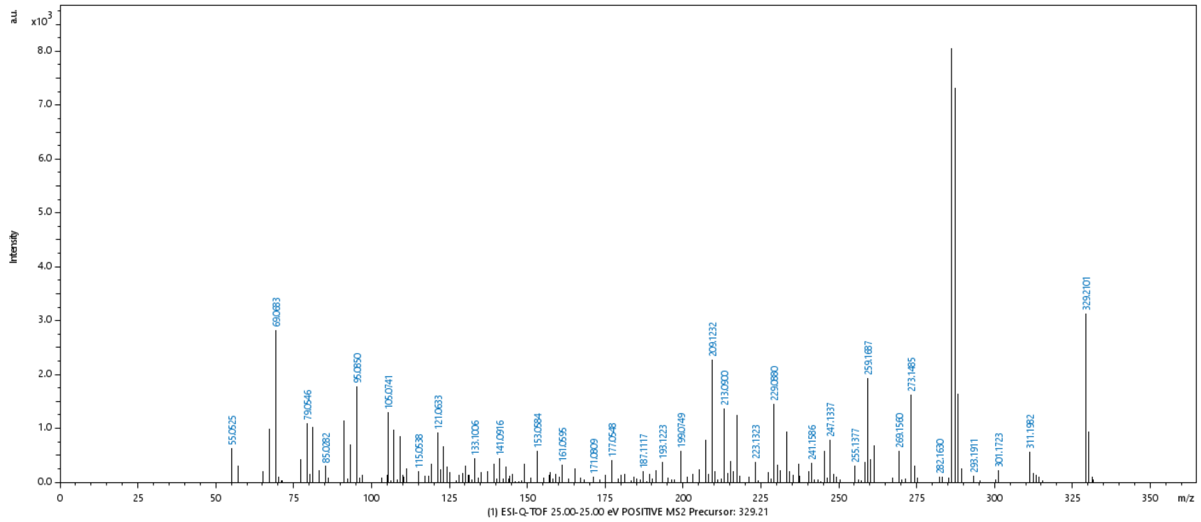

Figure S19. Experimental MS^2 fragmentation pattern of HU-331 standard.

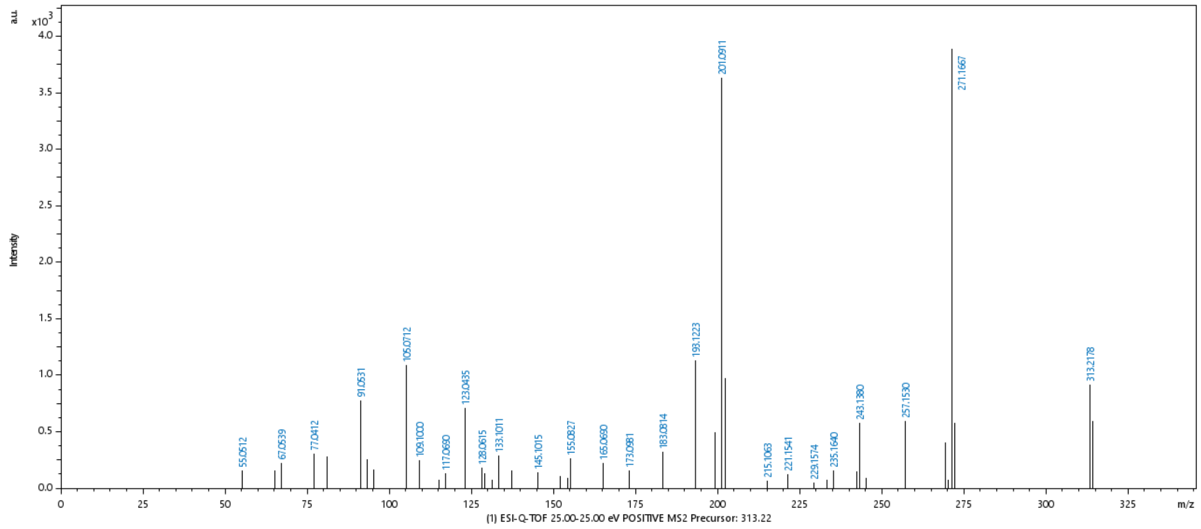

Figure S20. Experimental MS^2 fragmentation pattern of 6α-OH-CBD from CBD e-liquids at 1.84 min.

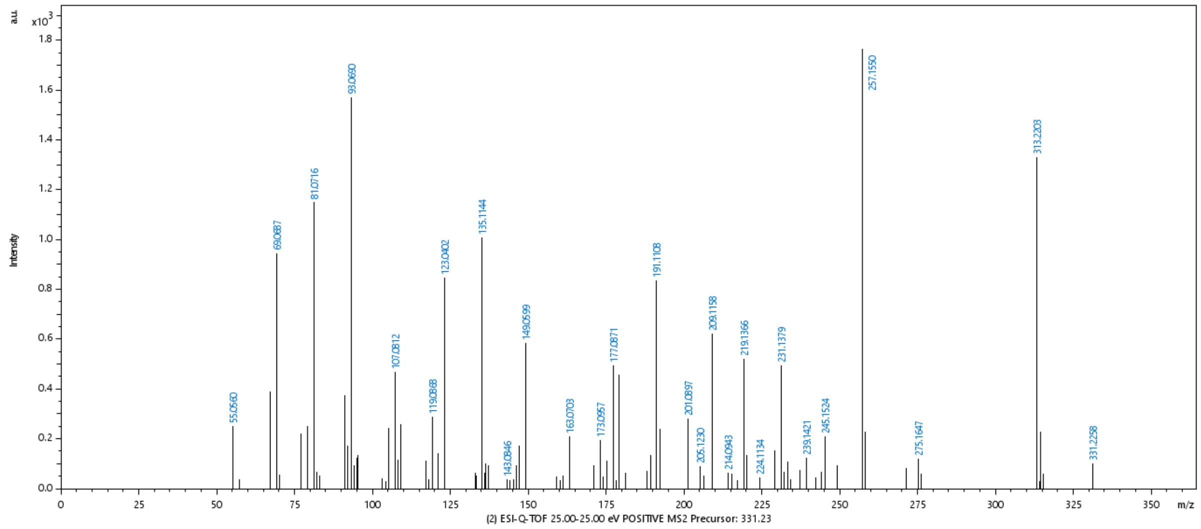

Figure S21. Experimental MS^2 fragmentation pattern of 7-OH-CBD from CBD e-liquids at 2.03 min.

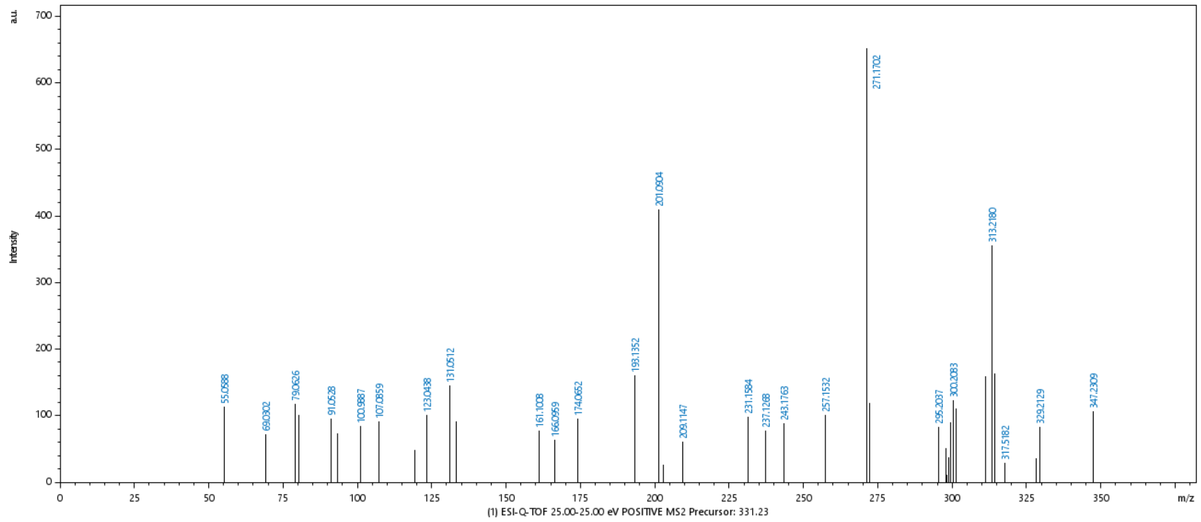

Figure S22. Experimental MS^2 fragmentation pattern of diOH-CBD from CBD e-liquids at 2.13 min.

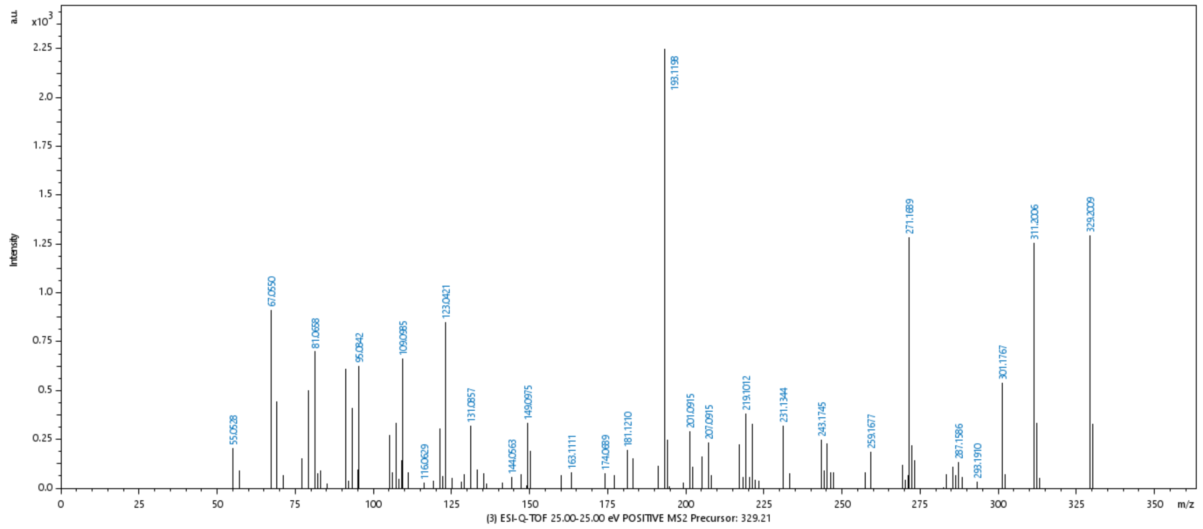

Figure S23. Experimental MS^2 fragmentation pattern of HU-331-like from CBD e-liquids at 2.42 min.

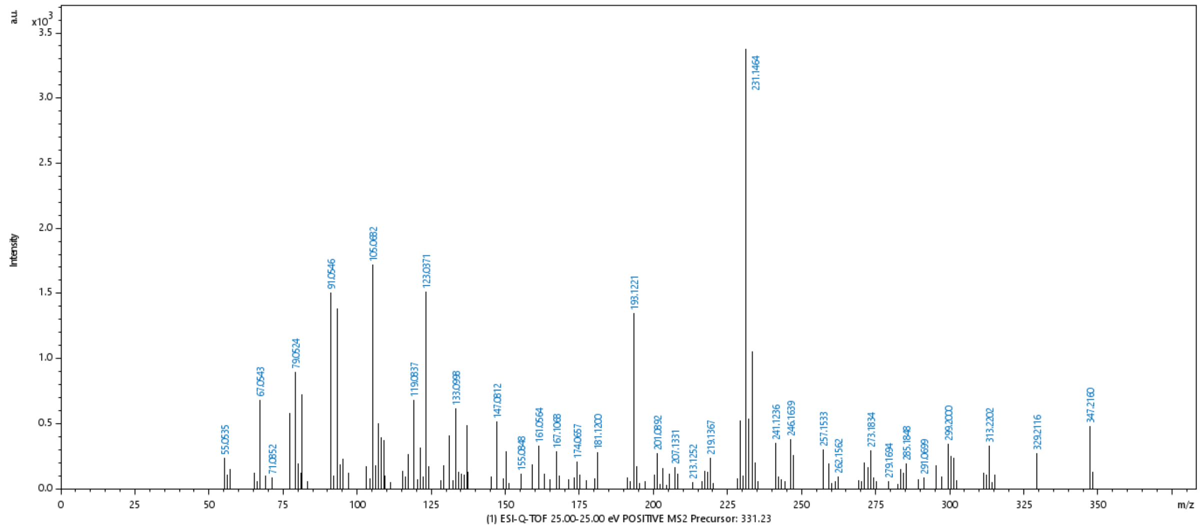

Figure S24. Experimental MS^2 fragmentation pattern of diOH-CBD from CBD e-liquids at 2.52 min.

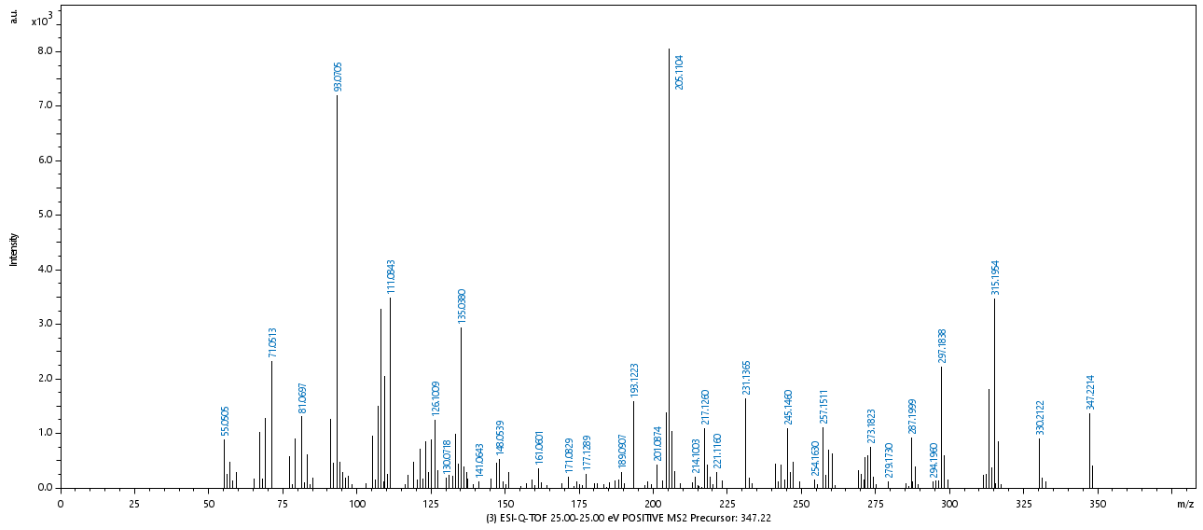

Figure S25. Experimental MS^2 fragmentation pattern of OH-CBE from CBD e-liquids at 2.76 min.

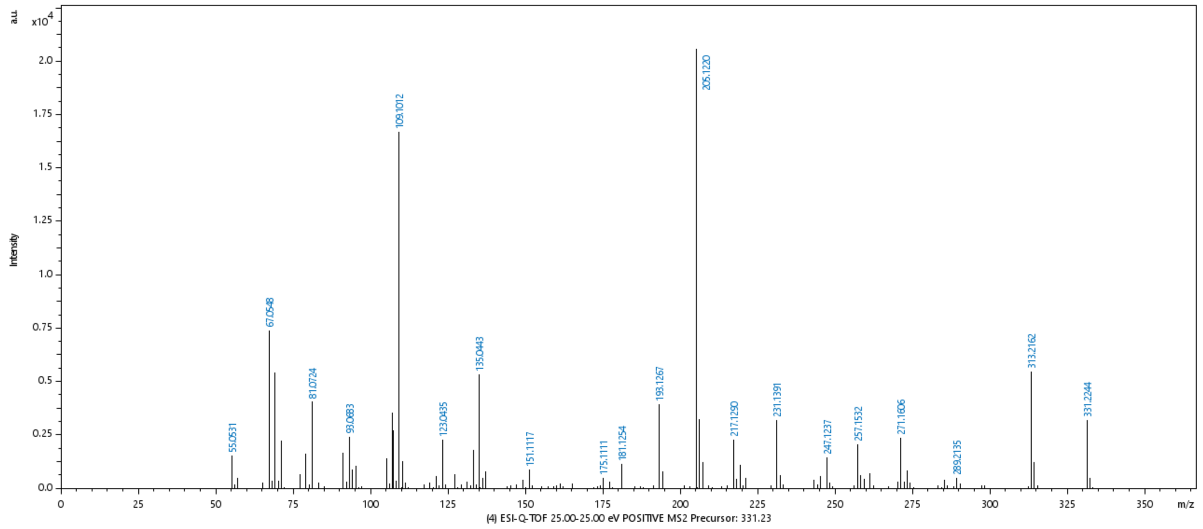

Figure S26. Experimental MS^2 fragmentation pattern of CBE isomer from CBD e-liquids at 2.85 min.

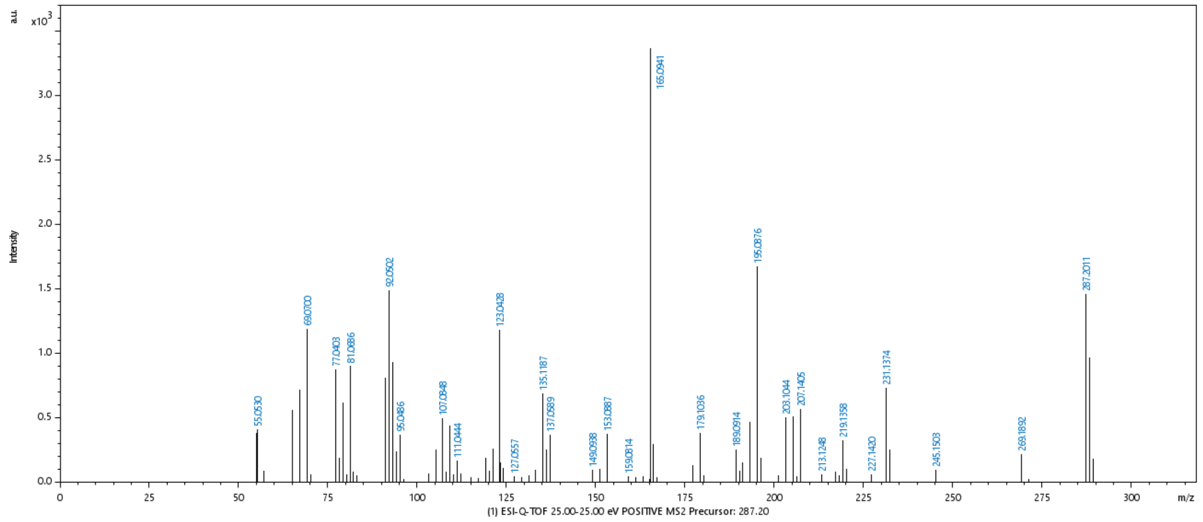

Figure S27. Experimental MS^2 fragmentation pattern of CBDV from CBD e-liquids at 3.20min.

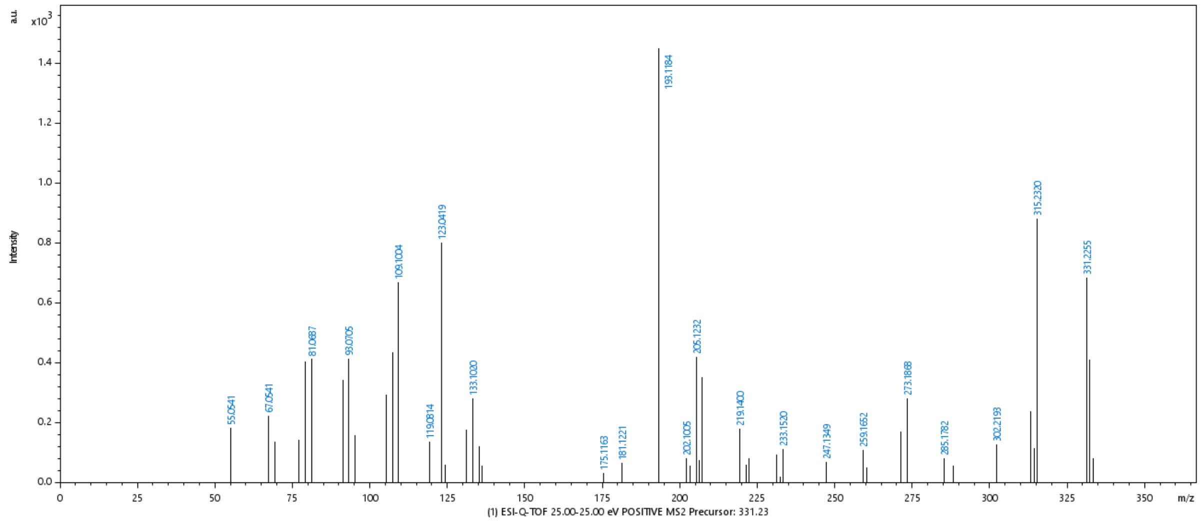

Figure S28. Experimental MS^2 fragmentation pattern of OH-CBD from CBD e-liquids at 3.22 min.

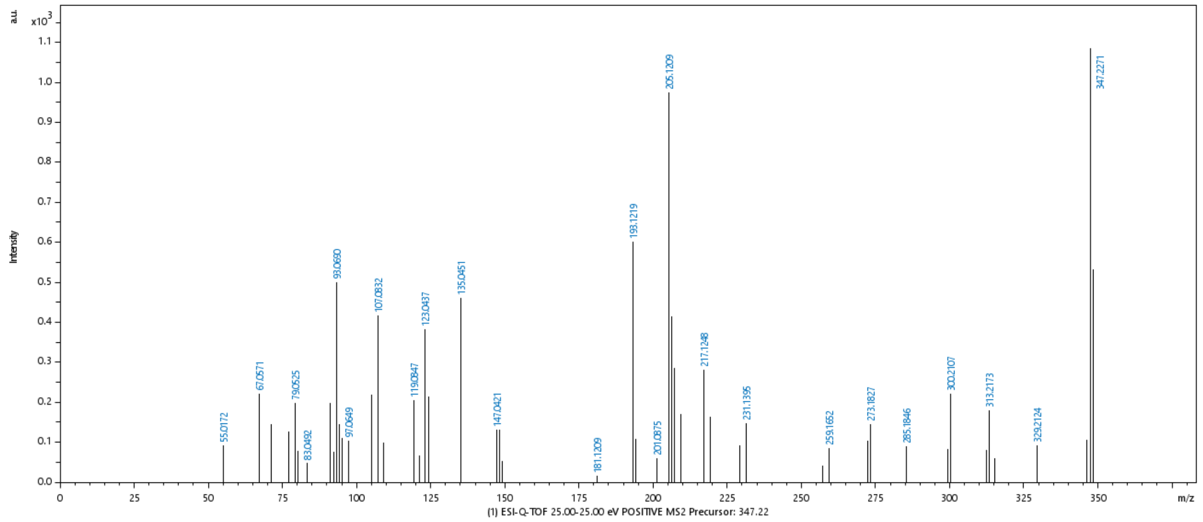

Figure S29. Experimental MS^2 fragmentation pattern of OH-CBE from CBD e-liquids at 3.61 min.

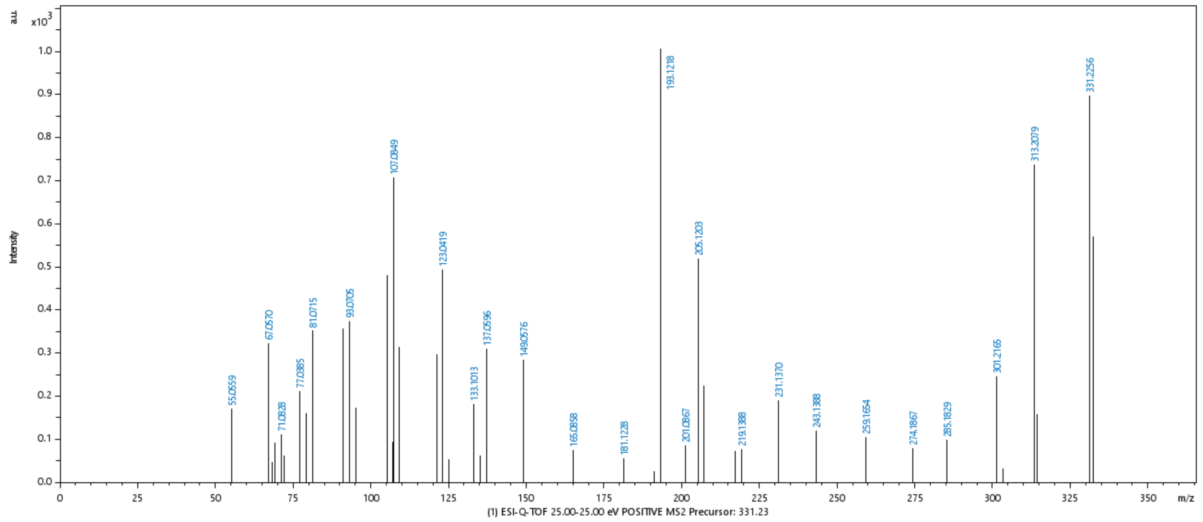

Figure S30. Experimental MS^2 fragmentation pattern of OH-CBD from CBD e-liquids at 3.67 min.

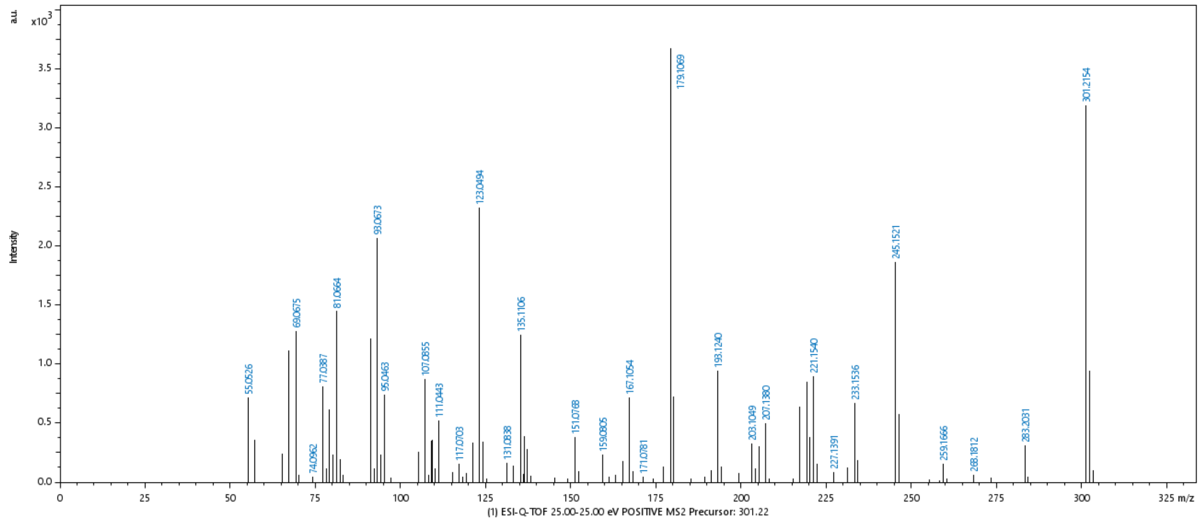

Figure S31. Experimental MS^2 fragmentation pattern of CBDB from CBD e-liquids at 3.92 min.

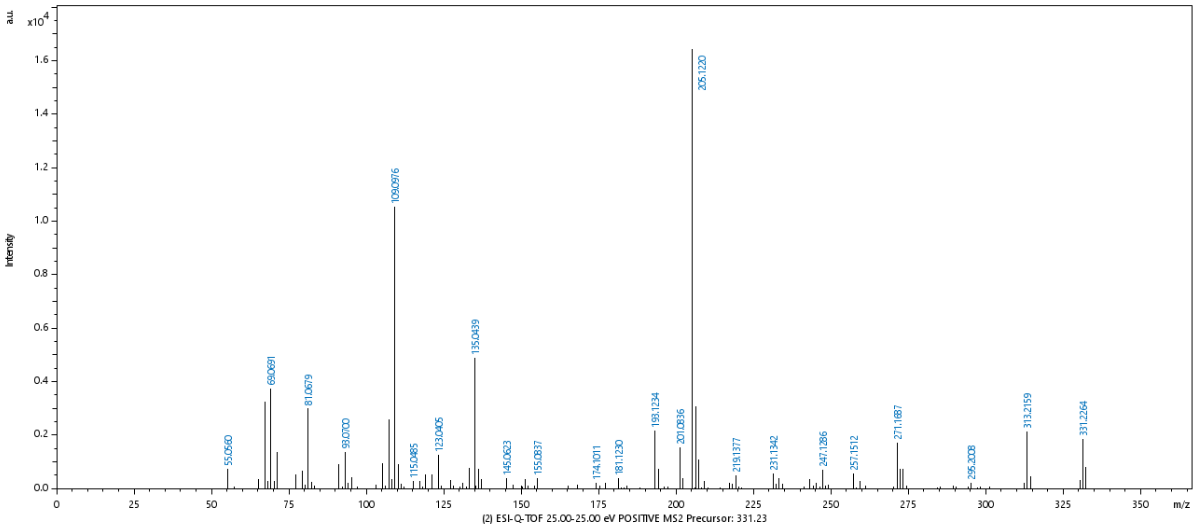

Figure S32. Experimental MS^2 fragmentation pattern of CBE from CBD e-liquids at 4.17 min.

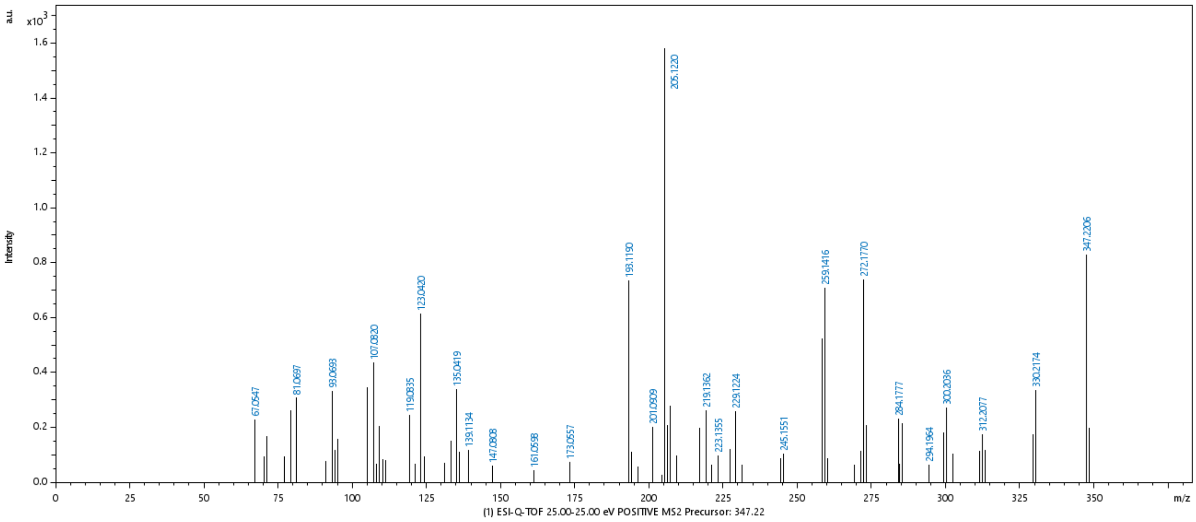

Figure S33. Experimental MS^2 fragmentation pattern of OH-CBE from CBD e-liquids at 4.21 min.

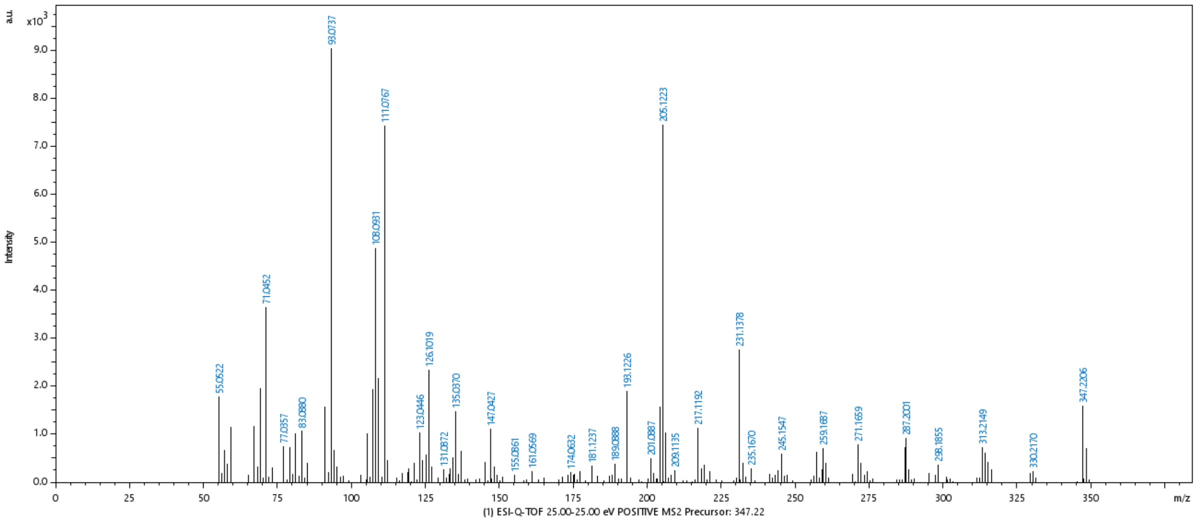

Figure S34. Experimental MS^2 fragmentation pattern of OH-CBE from CBD e-liquids at 4.75 min.

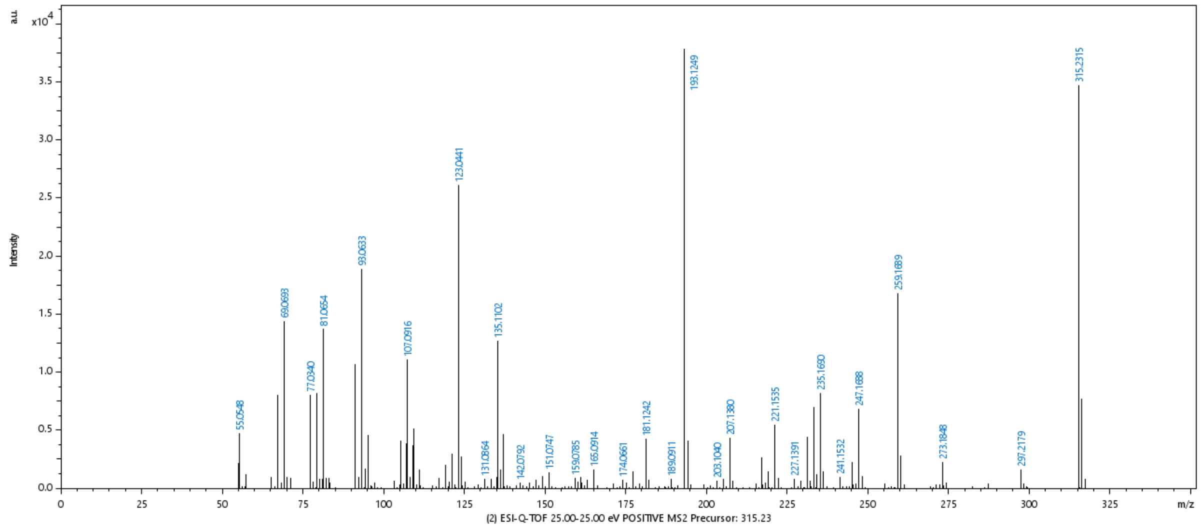

Figure S35. Experimental MS^2 fragmentation pattern of CBD from CBD e-liquids at 4.99 min.

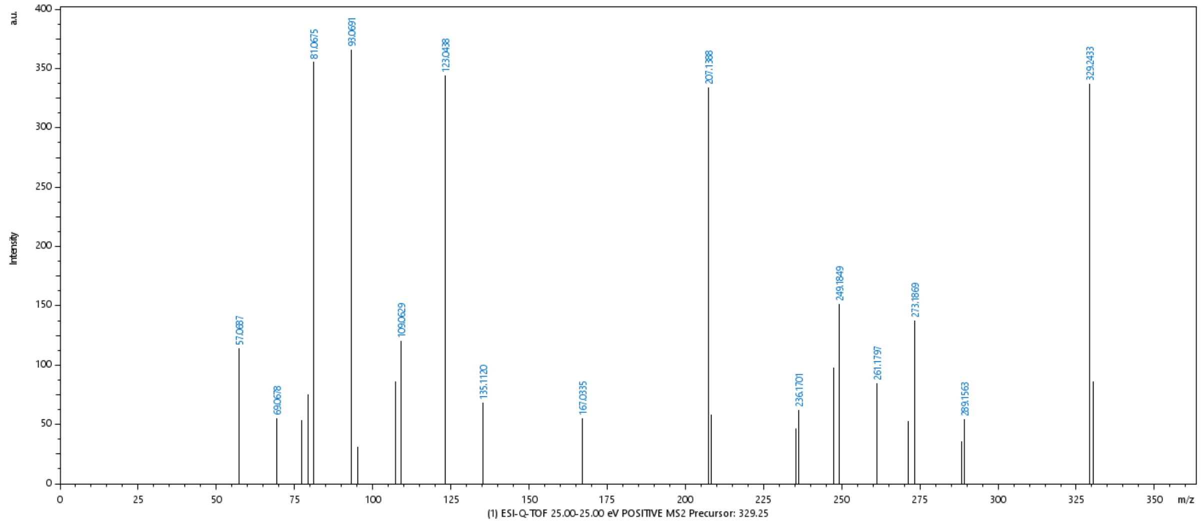

Figure S36. Experimental MS^2 fragmentation pattern of CBDH from CBD e-liquids at 6.56 min.

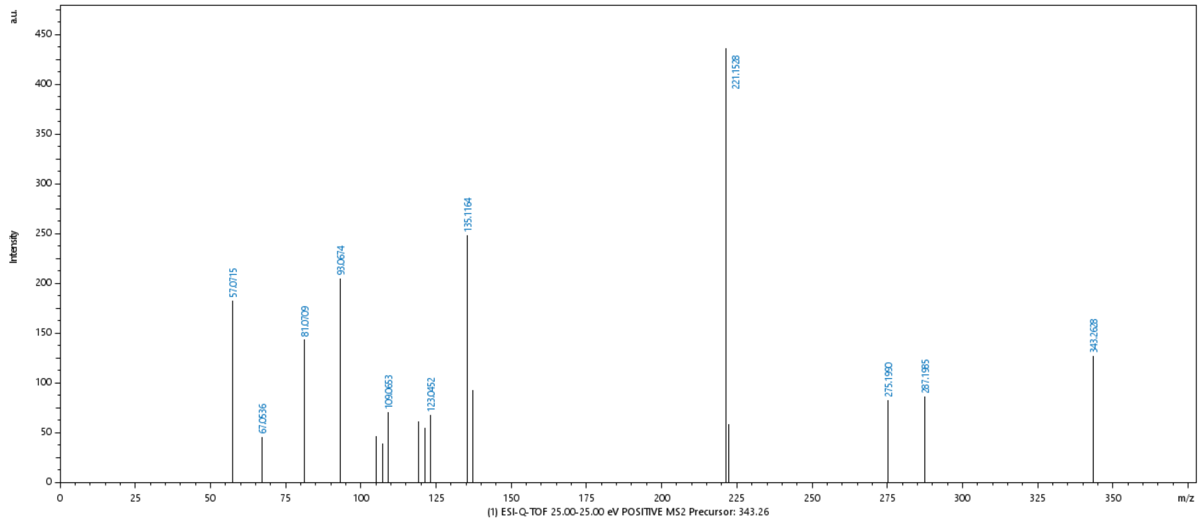

Figure S37. Experimental MS^2 fragmentation pattern of CBDP from CBD e-liquids at 8.95 min.

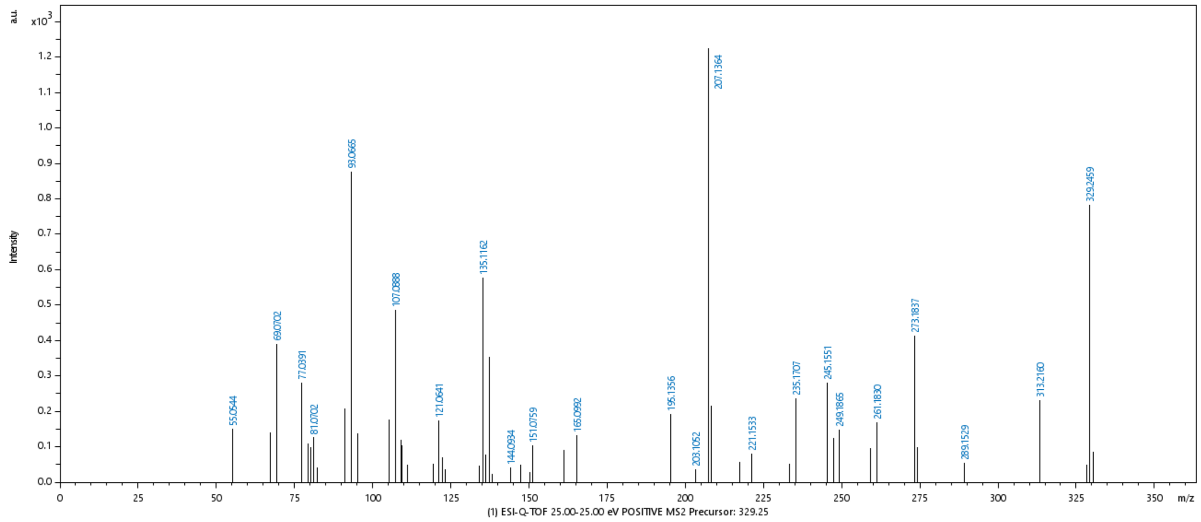

Figure S38. Experimental MS^2 fragmentation pattern of CBDH isomer from CBD e-liquids at 9.65 min.

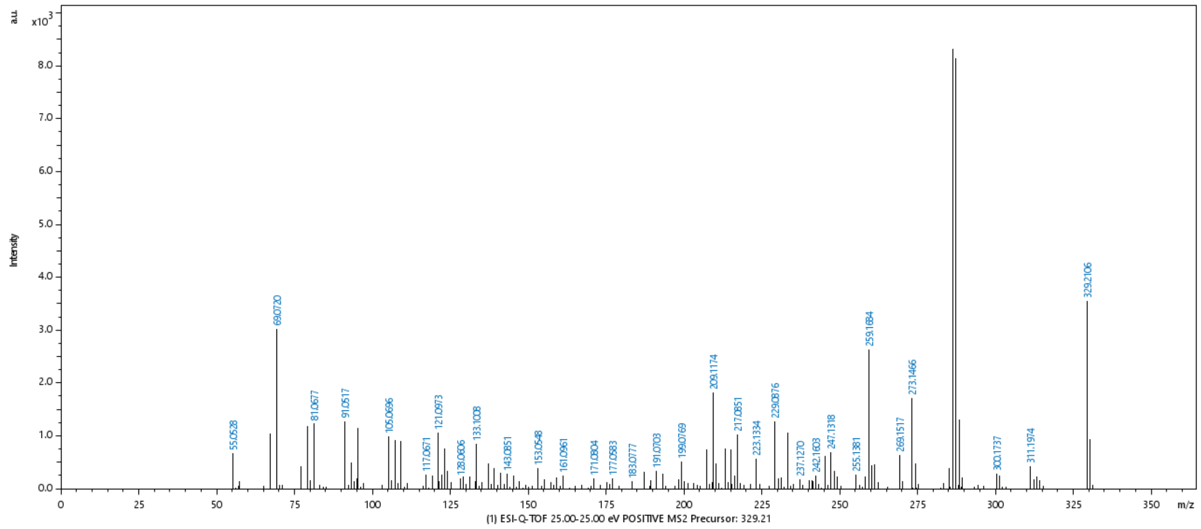

Figure S39. Experimental MS^2 fragmentation pattern of HU-331 from CBD e-liquids at 9.92 min.
